# Conformational energies of reference organic molecules: benchmarking of common efficient computational methods against coupled cluster theory

**DOI:** 10.1007/s10822-023-00513-5

**Published:** 2023-08-19

**Authors:** Ioannis Stylianakis, Nikolaos Zervos, Jenn-Huei Lii, Dimitrios A. Pantazis, Antonios Kolocouris

**Affiliations:** 1 Department of Medicinal Chemistry, Faculty of Pharmacy, National and Kapodistrian University of Athens, Panepistimioupolis Zografou, 15771 Athens, Greece; 2 Department of Chemistry, National Changhua University of Education, Changhua City, Taiwan; 3 Max-Planck-Institut für Kohlenforschung, Kaiser-Wilhelm-Platz 1, 45470 Mülheim an der Ruhr, Germany; 4 Laboratory of Medicinal Chemistry, Section of Pharmaceutical Chemistry, Department of Pharmacy, National and Kapodistrian University of Athens, Panepistimiopolis-Zografou, 15771 Athens, Greece

**Keywords:** Conformational energies, Force fields, DLPNO-CCSD(T), B3LYP, Organic molecules

## Abstract

We selected 145 reference organic molecules that include model fragments used in computer-aided drug design. We calculated 158 conformational energies and barriers using force fields, with wide applicability in commercial and free softwares and extensive application on the calculation of conformational energies of organic molecules, e.g. the UFF and DREIDING force fields, the Allinger’s force fields MM3-96, MM3-00, MM4-8, the MM2-91 clones MMX and MM+, the MMFF94 force field, MM4, ab initio Hartree–Fock (HF) theory with different basis sets, the standard density functional theory B3LYP, the second-order post-HF MP2 theory and the Domain-based Local Pair Natural Orbital Coupled Cluster DLPNO-CCSD(T) theory, with the latter used for accurate reference values. The data set of the organic molecules includes hydrocarbons, haloalkanes, conjugated compounds, and oxygen-, nitrogen-, phosphorus- and sulphur-containing compounds. We reviewed in detail the conformational aspects of these model organic molecules providing the current understanding of the steric and electronic factors that determine the stability of low energy conformers and the literature including previous experimental observations and calculated findings. While progress on the computer hardware allows the calculations of thousands of conformations for later use in drug design projects, this study is an update from previous classical studies that used, as reference values, experimental ones using a variety of methods and different environments. The lowest mean error against the DLPNO-CCSD(T) reference was calculated for MP2 (0.35 kcal mol^−1^), followed by B3LYP (0.69 kcal mol^−1^) and the HF theories (0.81–1.0 kcal mol^−1^). As regards the force fields, the lowest errors were observed for the Allinger’s force fields MM3-00 (1.28 kcal mol^−1^), ΜΜ3-96 (1.40 kcal mol^−1^) and the Halgren’s MMFF94 force field (1.30 kcal mol^−1^) and then for the MM2-91 clones MMX (1.77 kcal mol^−1^) and MM+ (2.01 kcal mol^−1^) and MM4 (2.05 kcal mol^−1^). The DREIDING (3.63 kcal mol^−1^) and UFF (3.77 kcal mol^−1^) force fields have the lowest performance. These model organic molecules we used are often present as fragments in drug-like molecules. The values calculated using DLPNO-CCSD(T) make up a valuable data set for further comparisons and for improved force field parameterization.

**Graphical abstract:**

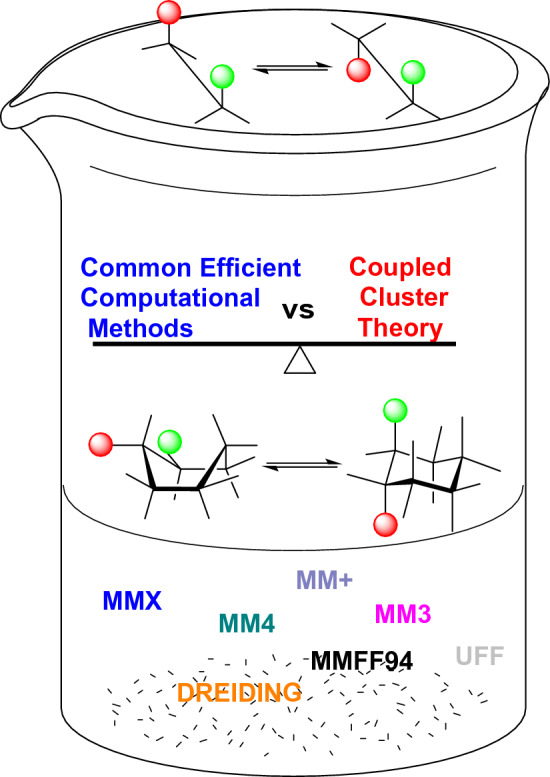

**Supplementary Information:**

The online version contains supplementary material available at 10.1007/s10822-023-00513-5.

## Introduction

Molecular mechanics methods are widely used for ranking and filtering huge numbers of organic molecules conformers, e.g., in structure-based drug design (SBDD) before docking calculations are applied [1–6], in ligand-based drug design (LBDD) [7–9], which is often followed by three-dimensional quantitative structure–activity relationships (3D-QSAR) predictions [10] or pharmacophore modeling [11]. Scoring functions based on force fields, e.g. MM3-96 [12] or semiempirical quantum mechanical (QM) methods, e.g., MNDO hamiltonians [13, 14], without or combined with an implicit solvent model [12, 14] are used for SBDD.

Conformational sampling using force fields of the drug molecules in SBDD problems can be accomplished using fragment-based approaches [15, 16], in which the candidate drug molecule is divided into fragments and the smaller organic molecule conformations are sampled before carrying out the docking calculations. Candidate conformations of the entire molecule are computed by re-combining favorable fragment conformations. A database of minimized conformations of the fragments allowed re-using them during conformer generation of other molecules, including drugs and large bioactive molecules, which improves the time-efficiency of sampling as, for example, in the open-access Quantum–Mechanical Properties of Drug-like Molecules (QMugs) data set; the QMugs collection comprises QM properties on optimized molecular geometries using *ω*B97X-D/def2-SVP, e.g., QM wave functions, including DFT density and orbital matrices, of more than 665 k biologically and pharmacologically relevant molecules extracted from the ChEMBL database, totaling ~ 2 M conformers [17, 18]. QM7-X [19] is another comprehensive dataset of 42 physicochemical properties for ~ 4.2 million equilibrium and non-equilibrium structures of small organic molecules with up to seven non-hydrogen optimized with PBE0 + MBD, i.e., a third-order self-consistent charge density functional tight binding (DFTB3) supplemented with a treatment of many-body dispersion (MBD) interactions. These methods use ensembles of conformations that capture the bioactive conformation as one of a diverse set of energetically accessible conformations [20–23]. Another approach involves using pre-existing knowledge of small-molecule conformations to further restrict the space of the conformational search to likely torsion angles and other combinations. Such knowledge-based methods [24, 25] derive torsion angle preferences from molecular mechanics or QM simulations of small molecules or structural databases such as the Cambridge Structure Database (CSD) [26] or the Protein Data Bank (PDB) [27]. Such datasets combined with ML can lead to the calculation up to 20 million off-equilibrium conformers of organic molecules [28].

Benchmarks of conformer generation tools have been performed, not based on low-energy/geometry [23], but also by comparing the geometry of an experimental crystal structure against an ensemble (e.g., 50 to 200+) of conformers [22, 29–35]; given a reasonable tool, one might guess that generating enough conformers should produce something close to the experimental geometry. Thus, finding a metric, such as energy, to filter, score or rank conformers is critical. Also, Bayesian optimization for conformer generation [36] or Graph Neural Network [37] have been applied to find the lowest energy conformer rather than an ensemble of conformers. Although most methods to score conformations, i.e., calculate conformational energies and identify low energy conformers, use a molecular mechanics energy function, e.g., the MMFF94 force field [38] implemented in MOE [39], in OMEGA program [9, 29], in RDKit [40], the OPLS2001 force field [41] implemented in the CONFGEN [42], the reliability of classical force fields and other quantum mechanical methods needs to be validated [43]. Other studies explored conformational search algorithms to regenerate bioactive conformations from protein–ligand-complexes. It was found that in 73% of the studied molecules in protein complexes from Protein Data Bank (PDB) structures the “bioactive” conformation was within 3*k*_B_T from the most-stable conformation in solution as determined by density functional theory (DFT) calculations [22].

Despite the enormous amount of energy values that can be produced with the current computing resources, systematic comparisons of force fields and QM methods provide always helpful results [44] for the calculation of the conformational energies of organic molecules [36, 45].

A multitude of studies benchmarked the ability of different force fields and QM methods in conformational analysis [46–59]. These studies evaluated the energies and conformations of organic molecules, including standard organic molecules, carbohydrates, amino acids, small peptides etc. and compare the force field and QM methods results to reference values, either from experiment or ab initio calculations [46–59]. Examples are the evaluation, e.g., of Allinger MM2 force field and its clones, MM3-96, MMFF94, MMFF94s [48, 50], of Tripos and MMFF94 force fields  [32], OPLS3, OPLS-2005, MMFFs and AMBER* force fields [34], MMFF94 (before and after reparameterization of torsion angles with MP2/cc-pVTZ), OPLS3, MM2 force fields [35], or force fields (e.g., OPLS-AA, MMFF, CHARMMm) and QM methods (i.e. B3LYP, M06-2X) [33] or the *E*/*Z* energetics for molecular modeling [60]. A review that described these efforts in some detail has been published [61]. The DFT single-point energies [58] or MMFF94 force field energies were tested in reproducing the domain-based local pair natural orbital coupled-cluster theory [DLPNO‐CCSD(T)] with cc‐pVTZ basis set conformational energies and spatial structures for 37 organic molecules representing pharmaceuticals, drugs, catalysts, synthetic precursors, and industry‐related chemicals (37conf8 database) [36]. The DLPNO approximation has enabled the first CCSD(T) calculation of a protein containing 644 atoms [62], model systems of enzymatic reactions [63], conformational energies [64–66] and in benchmarking of the GMTKN55 superset of molecules that contains 1505 relative energies and 2462 single-point calculations [67]. Additionally, a benchmark study on the performance of force field, semiempirical, and DFT methods for the calculation of relative conformer energies for 100 compounds of lead-like and drug-like was performed using the DFT PBE0-D3(BJ)//def2-TZVP energy values as reference [68]. A comparison of computational methods force fields, DFT functionals, and machine learning potentials have been performed in a set of 700 molecules; 10 conformations of each molecule were chosen and optimized at B3LYP-D3BJ/def2-SVP level of theory followed by single-point energy calculations at the “gold standard” DLPNO-CCSD(T)/cc-pVTZ level of theory [59]. Local minima geometries across ~ 700 molecules drug-like molecules, have been repeatedly used to evaluate the quality of conformer generation [9, 69], each optimized by B3LYP‐D3(BJ) with single‐point DLPNO‐CCSD(T) triple‐zeta energies. The 6500 single points generated were compared with results from the application of force fields, semiempirical hamiltonians, DFT functionals and machine learning (ML) methods for conformational energies ranking of minima [45].

Given that drug molecules consist of a few tens of atoms connected by covalent bonds and that the possible organic small molecules number hundreds of billions [70], hardware progress can lead to the accurate coupled cluster method with single-, double-, and perturbative triple excitations [CCSD(T)] chemical energies for 133,000 molecules with less than 10 carbons [71], or B3LYP/6-31G* optimized geometries for 2.6 million molecules [72].

Continued development of deep learning molecular potentials generated from QM data sets can provide high accuracy predictions of QM reference calculations, while maintaining a computational cost comparable to classical force field. ANI-2x potential provided chemically accurate energy predictions for molecules containing seven atomic elements (H, C, N, O, S, F, Cl) of interest to computational drug design (CADD) and showed similar accuracy to DFT methods, while outperformed MMFF94 and PM6 for conformer scoring. The ANI-2x potential retained the same computational scaling as classical force fields, providing a 10^6^ speedup over the DFT level it has been trained against [73]. It has been tested across a wide range of applications relevant to drug development on diverse test sets.

For drug design purposes the accurate description of the diverse local minima is needed for each drug-like molecule [74], and datasets have been developed towards that aim, e.g. the platinum dataset of 2912 protein-bound ligand conformations extracted from the PDB for which the chemical space was shown to be representative of the chemical space of approved drugs [75].

In the present work we seek to revisit results of calculations for model organic molecules of standard conformational interest rather than drug-like compounds and focuses on assessing the accuracy of force fields, frequently used in conformer search applications, but also standard ab initio and DFT methods. Compared to previous works, e.g. in Refs. [48, 50] we increased the number of tested molecules and the number of methods applied, which include many force field methods but also Hartree–Fock (HF) theory, the post-HF second-order Møller–Plesset perturbation theory (MP2) and the standard DFT functional B3LYP. Accurate reference values for evaluating all these methods were obtained with the gold-standard basis-set extrapolated DLPNO-CCSD(T) method [76], in contrast to previous works which often used inconsistent experimental values or low theory levels as reference values. The suitable energies for comparison with CC-calculated conformational energies are energies measured in the gas phase with spestroscopic methods. Compared to previous works, this manuscript also reports the gas phase experimental and calculated with various theories conformational energies and describes the molecular basis of these conformational preferences for the organic molecules tested. These model organic molecules are often present as fragments in drug-like molecules. The values calculated using DLPNO-CCSD(T) make up a valuable data set for further comparisons and for improved force field parameterization.

## Computational methods

### Test set

A data set consisting of 158 small molecules consisting solely of carbon, hydrogen, nitrogen, and oxygen (CHON) atoms, was the same as that used before for validation of the accuracy of MMFF94 force field and subset of these has been previously used [48, 50]. The three dimension (3D) structures of the compounds used are available following the link found in the Supporting Information.

### Details for the calculations

Molecular mechanics calculations with the MM+ force field were performed using the Hyperchem program (Hypercube Inc.) which provided enthalpic values. For each molecule an initial structure was constructed and minimized first using a first derivative algorithm (conjugate gradient or steepest descents) and then Newton–Raphson algorithms and an energy gradient tolerance of 0.001 kcal mol^−1^ Å^−1^. HyperChem provides a variety of tools for the convenient manipulation of 3D structures like changing chirality, reflection through planes, easy insertion of a variety of torsional restraints etc. When a constraint was required the force constant used was as high as 150 kcal mol^−1^ degree^−2^. MMX, MM3-96 [77–79] and MMFF94 [32] were implemented with PCMODEL9/Windows software (Serena Software) and calculated enthalpies. 3D structures were minimized first using steepest descents and then Newton–Raphson algorithms and an energy gradient tolerance of 0.0001 kcal mol^−1^ Å^−1^. MM3-00 and MM4-08 [80–82] were performed using the commercial programs developed by Allinger and we calculated enthalpies [83, 84]. Calculations were carried out in the gas phase using a dielectric value of 1.5 and no cutoff for the nonbonded interactions. In the case of MM+ [85] available with Hyperchem program and MMX, which are clones of MM2-91 [86] force field, lone pair (lp) pseudo-atoms were added where needed for proper description of the molecular structure. Calculations of enthalpic values with UFF [87], Dreiding [88] force fields were carried out with Gaussian-03 software [89]. MM+, UFF and Dreiding are universal force fields and can be used for the calculations of any structure since, in cases for which no parameters are available, empirical rules are applied. Structure manipulations, restraints, etc. were applied using the software tools. In a few cases, structure manipulation was performed using HyperChem because this software is user friendly. The structures were then saved in PDB format and opened with other software pieces. B3LYP/6-31G(d,p) and MP2/6-31G(d,p) electron energy calculations were performed using Gaussian-09 [89] and the energies were calculated in geometry-optimized structures. ^−^

The B3LYP/6-31G(d,p) geometry-optimized structures were used for the DLPNO-CCSD(T) electronic energies calculations. Reference values for the electronic energies of all species were obtained at the CCSD(T) level [76] with separate extrapolation of the Hartree–Fock (HF) and electron correlation energies to their complete basis set (CBS) limits:

$${{E}_{\mathrm{total},\mathrm{ CCSD}(\mathrm{T})}^{\mathrm{CBS}}={E}_{\mathrm{HF}}^{\mathrm{CBS}}+E}_{\mathrm{corr}}^{\mathrm{CBS}}$$

The domain-based local pair natural orbital (DLPNO) methodology enabled the use of large correlation-consistent basis sets of polarized triple-zeta quality in the coupled-cluster calculations for all molecules included in this study. CBS extrapolation used correlation-consistent basis sets cc-pV*n*Z with successive cardinal values *n* = 2 and 3 (cc-pVDZ and cc-pVTZ) [90–92]. DLPNO-CCSD(T) calculations with the cc-pVQZ basis sets were not possible for all compounds due to memory limitations; therefore, for consistency, only cc-pV[D/T]Z extrapolated results will be discussed. Using a two-point exponential extrapolation [93, 94], the HF energy has been suggested to converge as:

$${E}_{\mathrm{HF}}^{\mathrm{CBS}}=\frac{{E}_{\mathrm{HF}}^{(X)}{e}^{(-\alpha \sqrt{Y})}-{E}_{\mathrm{HF}}^{(Y)}{e}^{(-\alpha \sqrt{X})}}{{e}^{(-\alpha \sqrt{Y})}-{e}^{(-\alpha \sqrt{X})}}$$

where* X* and *Y* represent the successive cardinal numbers of the basis sets. ${E}_{\mathrm{HF}}^{(X)}$ and ${E}_{\mathrm{HF}}^{(Y)}$ are the SCF energies obtained with the two basis sets. The parameter *α* takes the value of 4.42 for the cc-pV[D/T]Z extrapolation [94]. On the other hand, the CBS limit for the CCSD(T) correlation energy was obtained as:

$${E}_{\mathrm{corr}}^{\mathrm{CBS}}=\frac{{X}^{\beta }{E}_{\mathrm{corr}}^{(X)}-{Y}^{\beta }{E}_{\mathrm{corr}}^{(Y)}}{{X}^{\beta }-{Y}^{\beta }}$$

Here *X* and *Y* are the cardinal numbers as above and the optimal value for the parameter *β* for the cc-pV[D/T]Z combination of basis sets was shown to be 2.46 [94, 95]. The SCF component of the calculations employed the RI-JK approach in conjunction with cc-pVTZ/JK basis sets, while the cc-pVDZ/C and cc-pVTZ/C basis sets were used in the correlation treatment [96]. The ORCA program package was used for all DLPNO-CCSD(T) calculations [97]. TightSCF and TightPNO settings as defined in ORCA were used for all calculations. PNO extrapolation using different TCutPNO values was also tested [98–101], but it made no difference compared to the standard TightPNO computed energies for the molecules in the test set, confirming that the DLPNO values are converged with respect to the PNO space.

## Results and discussion

### Methods used and tested compounds

Tables 1, 2, 3, 4, 5, 6, 7, and 8 shows the results for conformational energies in structures whose geometry was optimized with force fields. The B3LYP/6-31G(d,p) and MP2/6-31G(d,p) energies were calculated in the geometry-optimized structures at the same level of theory. The B3LYP/6-31G(d,p) geometry-optimized comformations were used for the DLPNO-CCSD(T)/cc-pV[D/T]Z calculations procedure, which also provided the conformational energies for HF theory with different basis sets. DLPNO‐CCSD(T) performs exceedingly well in calculating the enthalpies of formation for molecules containing the elements H, C, N, O, F, S, Cl, Br [102]. It is noted that comparisons of DLPNO-CCSD(T) with cc-pVDZ and cc-pVTZ values for several representative molecules of all sub-groups showed that the difference in relative energies is typically of the order of 10^−2^ kcal mol^−1^. Previous works used ambiguous reference conformational energy values, obtained either from experiments performed in various conditions (see notes in Tables 1, 2, 3, 4, 5, 6, 7, and 8) or by employing low theory levels (see, for example, the conformation for tetrabenzylethene, 1,2-dicyanoethane, vinyl alcohol, glycolic acid, methyl glycolate dimethoxymethane, methylethylamine, hexahydropyrimidine, 3-OH-hexahydro-pyrimidine, ethyldimethyl ammonium, tropane, 3-OH-hexahydro-pyrimidine, *N*-methylamide, *N*-acetylalanine, *N*-acetylphenylalaninyl-amide, methyformate, phosphine, methylethylsulfone, sulfolane, 2-methylpropenal, but-1-ene-3-one, and the rotational barriers for methyl formate, dimethyl phosphine, and trimethyl-phosphate in Refs. [48, 50, 82, 103–111]. Compared to previous works, in the present study we calculated the relative energies at the DLPNO-CCSD(T) level of theory [76] with cc-pV[D/T]Z CBS extrapolation and used them as reference conformational energy values. In the relevant tables we also included experimental values previously obtained and used for some of these molecules for comparative purposes. We note, however, that these are not always directly comparable because the computed energies reported here are electronic gas-phase values and do not incorporate thermodynamic or solvent contributions [48]. In the tables, when a method performs with an error larger than 1.5 kcal mol^−1^, the result is indicated in boldface and bold underlined when the deviation is larger than 3 kcal mol^−1^. When a conformational energy difference with a tested theory has opposite sign compared to the reference theory but the energy value differs by less than ~ 0.1 kcal mol−1, we considered this case as a correct prediction with the tested theory.

**Table 1:** Relative conformational energies of few hydrocarbon molecules (kcal mol^−1^)

Compound	Description	UFF	DREIDING	MM+	MMX	MMFF94	MM3-96	MM3-00	MM4-08	HF/cc-pVDZ	HF/cc-pVTZ	HF/CBS	B3LYP/6-31G(d,p)	MP2/cc-pVTΖ	DLPNO-CCSD(T)	Expt
*n*-Butane	*g-a*	1.11	0.77	0.86	0.86	0.78	0.81	0.81	0.68	1.05	1.06	1.06	0.91	0.63	0.60	0.67–0.7^a^
1-Butene	*Cis-skew*^e^	− 3.55	**2.30**	0.49	0.49	0.26	0.68	0.72	0.64	0.54	0.61	0.63	0.47	0.14	− 0.01	0.47^a^^,^^b^
2,3-Dimethylbutane	*g-a*	− 0.50	− 1.15	0.15	0.15	− 0.23	0.39	0.38	0.14	− 0.15	− 0.07	− 0.05	0.04	0.14	0.16	0.05^a^
Tetraethylmethane	T3-T1	− 0.03	0.23	0.16	0.16	0.45	0.21	0.20	0.19	0.86	0.87	0.88	0.82	1.08	1.01	0.79^d^
Tetramethylhexane	C_2_-C_2h_	0.13	0.15	0.05	0.05	0.18	0.13	− 0.41	− 0.35	− 0.01	− 0.01	0.00	0.00	0.01	− 0.01	0.22^d^
1,2-Diphenylethane	*g-a*	− 1.39	**− 2.31**	0.95	**2.91**	0.08	0.44	0.31	0.05	0.89	1.11	1.18	0.86	− 0.99	− 0.32	**1.19**^c^
Tetrabenzylethene	*C*_2*h*_-*D*_2_	− 0.65	1.06	**− 2.51**	**2.72**	**2.56**	1.81	0.35	1.16	7.05	6.91	6.87	3.05	3.58	4.25	–
2,4,6-Tribromo-1,3,5-trineopentylbenzene	*Twosyn-allsyn*	4.92	4.08	**1.55**	**1.50**	**2.24**	**2.24**	1.33	1.30	0.82	0.79	0.78	2.62	**1.57**	1.45	1.04^d^
Cyclohexane	*Twb-ch*	8.85	7.70	5.35	5.36	5.93	5.76	5.76	6.27	7.07	6.83	6.75	6.43	6.28	5.97	5.50^a^
Cyclohexene	*Boat-half ch*	5.91	5.30	6.07	6.10	7.60	6.67	6.58	7.27	6.86	6.67	6.61	5.48	5.51	5.54	5.50^d^
Cyclooctane	*TCC-BC*	*− *2.42	− 0.02	0.97	0.97	1.44	1.11	1.12	1.11	0.34	0.28	0.26	0.39	**2.29**	**1.97**	1.90^d^
**Νο. wrong conformers**		5	4	4	3	4	3	2	2	3	3	3	3	2		
**RMSD**		2.68	1.80	2.13	1.19	0.90	0.98	1.31	1.16	1.21	1.17	1.17	0.82	0.32		

^a^Δ*Η*, gas phase

^b^Δ*H*, solution, Raman

^c^Δ*G*, gas phase

^d^Δ*G*, solution, low temp. NMR

^e^Refers to C=C-C-C dihedral

**Table 2:** Relative conformational energies of some haloalkanes (kcal mol^−1^)

Compound	Description	UFF	DREIDING	MM+	MMX	MM3-96	MMFF94	MM3-00	MM4-08	HF/cc-pVDZ	HF/cc-pVTZ	HF/CBS	B3LYP 6-31G(d,p)	MP2/cc-pVDZ	DLPNO-CCSD(T)	Expt.
CH_3_CH_2_ CH_2_F	*g-a*^b^	0.26	0.21	0.08	0.08	0.20	− 0.06	− 0.18	− 0.22	− 0.31	− 0.08	0.0	− 0.10	− 0.26	− 0.19	− 0.35^a^
CH_3_CH_2_ CH_2_Cl	*g-a*^b^	0.77	0.52	0.21	0.24	0.14	− 0.01	0.14	− 0.13	0.14	0.35	0.41	0.20	− 0.04	− 0.02	− 0.09^a^
FCH_2_ CH_2_F^a^	*g-a*^b^	0.05	0.08	− 0.64	0.50	− 0.23	− 0.63	− 0.19	− 0.66	0.23	− 0.24	− 0.40	− 0.66	− 0.77	− 0.78	− 0.56^a^
ClCH_2_ CH_2_Cl^a^	*g-a*^b^	0.59	0.40	**1.63**	**1.96**	**2.30**	1.24	1.03	1.35	**1.86**	**1.92**	**1.94**	**1.68**	1.44	1.47	1.05^a^
ClCH_2_CH_2_ CH_2_Cl^a^	*aa-g*^+^*g*^+c^	**− 1.60**	− 1.00	0.13	0.70	0.80	1.13	0.41	0.86	1.40	0.93	0.77	1.22	1.70	**1.58**	1.09^a^
CH_3_CH_2_ CH_2_F	*ag-g*^+^*g*^+c^	− 0.83	− 0.49	− 0.01	0.19	0.30	0.39	0.14	0.42	0.69	0.47	0.40	0.63	0.95	0.90	0.78^a^
**Νο. wrong conformers**		5	5	3	3	2	0	1	0	2	1	2	1	0		
**RMSD**		0.74	1.60	0.72	0.72	1.35	0.60	0.30	0.64	0.06	0.47	0.46	0.36	0.23		

^a^Δ*Η*, gas phase

^b^*α*: in this *Newman* projection the torsion angle Χ-C-C-C or Χ-C-C-X is ~ 180°, *g*: the relative torsion angle is ~ + 60° or − 60°

^c^*aa*: the torsion angle between Cl-C1-C2-C3 is ~ 180°, *g*^+^*g*^+^: the torsion angle between Cl και C-3 is rotate in a clockwise direction by ~ + 60°, *ag*: one torsion angle between Cl and C-3 is ~ 180° and the other is ~ + 60 or − 60° [112]

**Table 3:** Relative conformational energies of some cyclohexane derivatives (kcal mol^−1^)

Compound	Description	UFF	DREIDING	MM+	MMX	MM3-96	MMFF94	MM3-00	MM4-08	HF/cc-pVDZ	HF/cc-pVTZ	HF/CBS	B3LYP 6-31G(d,p)	MP2/cc-pVDZ	DLPNO-CCSD(T)	Expt. or high level theory^e,f^
Methylcyclohexane	ax-eq	**1.87**	1.29	**1.78**	**1.78**	**1.78**	1.37	**1.77**	**1.51**	**2.44**	**2.47**	**2.48**	**2.28**	**1.76**	**1.71**	1.76^b^
*i*-Pr-cyclohexane	ax-eq	**2.10**	**1.65**	**1.72**	**1.72**	**1.86**	**1.53**	**1.86**	**1.55**	**2.44**	**2.50**	**2.51**	**2.26**	1.28	1.37	1.40^b^
*t-*Bu-cyclohexane	ax-eq	8.93	6.29	5.51	5.00	6.14	6.21	6.20	5.63	6.44	6.40	6.39	5.46	5.27	5.10	4.90^c^
Phenylcyclohexane	ax-eq^g^	6.65	**2.83**	3.41	3.46	4.07	**2.58**	4.04	**2.14**	4.44	4.60	4.65	3.99	**2.69**	**2.73**	2.87^c^
Me_3_Si-cyclohexane	ax-eq	5.23	5.18	**2.60**	**2.88**	**2.95**	**2.04**	**2.90**	**2.82**	3.45	3.59	3.64	3.20	**2.39**	**2.44**	2.50^d^
Cyclohexylamine	ax-eq	0.83	0.48	1.40	1.40	1.22	0.67	1.23	*3.38*	0.62	1.09	1.24	1.26	0.53	0.74	1.49^c^
Cyclohexanol	ax-eq	0.62	0.24	0.59	0.59	0.72	0.32	0.74	0.46	0.20	0.64	0.78	0.88	0.27	0.44	0.52^c^
Methoxycyclohexane	ax-eq	0.58	0.28	0.55	0.55	0.75	0.41	0.76	0.11	0.09	0.55	0.71	0.69	− 0.08	0.15	0.55^c^
Me_3_SiO-cyclohexane	ax-eq^h^	0.38	0.18	0.26	0.30	0.60	0.30	0.57	0.00	− 0.39	− 0.02	0.10	0.48	− 0.68	− 0.44	**1.31**^c^
Ph_3_SiO-cyclohexane	ax-eq^h^	0.51	− 0.08	0.20	0.25	0.50	0.16	0.51	− 0.07	0.82	1.24	1.38	1.15	− 0.12	0.04	0.71^c^
Cyclohexylmethylketone	ax-eq	**2.60**	**2.18**	1.31	1.29	1.56	− 0.21	**1.60**	1.43	1.22	1.48	**1.56**	1.41	0.41	0.59	1.17^b^
Methyl cyclohexanecarboxylate	ax-eq	**1.69**	**1.53**	1.33	1.25^b^	1.32	− 0.38	1.38	1.33	0.68	1.40	**1.63**	**1.57**	0.49	0.69	1.12^b^
Cyclohexanethiol	ax-eq	1.39	0.95	1.08	1.20	n.d.	0.13	1.49	0.68	1.44	**1.79**	**1.91**	**1.68**	0.98	1.03	~ 1.10^c^
Cyclohexylphosphine	ax-eq	− 0.47	− 0.42	0.65	n.d.	1.45	0.65	1.43	0.96	**2.11**	**2.37**	**2.46**	**2.14**	1.49	**1.52**	1.6^c^
Fluorocyclohexane	ax-eq	0.39	0.34	0.15	0.16	0.25	− 0.37	− 0.49	0.11	− 0.24	0.16	0.29	0.31	0.08	0.14	0.16^a^
Chlorocyclohexane	ax-eq	1.19	0.82	0.42	0.43	0.23	− 0.35	0.33	0.29	0.59	0.99	1.12	0.98	0.41	0.40	0.50^a^
*Trans*-1,2-dimethylcyclohexane	ax,ax-eq,eq	**1.93**	1.35	**2.43**	**2.43**	**2.57**	**1.80**	**2.57**	**2.18**	3.32	3.41	3.44	3.22	**2.54**	**2.49**	2.58^c^
*Cis*-1,3-dimethylcyclohexane	ax,ax-eq,eq	6.69	4.63	5.34	5.34	5.72	5.08	5.70	4.99	6.75	6.81	6.83	6.16	5.31	5.08	5.50^a^
*Trans*-1,2-bisSiMe_3_-cyclohexane	ax,ax-eq,eq	**− 2.25**	*− *8.46	**− 1.87**	**− 1.51**	− 1.25	*− *4.93	− 1.16	− 0.95	− 1.78	**− 1.83**	**− 1.84**	− 1.04	− 0.38	− 0.33	~ − 1.60^c^
*Trans*-1,2-difluoro-cyclohexane	ax,ax-eq,eq	0.73	0.61	0.82	− 0.57	− 0.18	− 0.22	− 1.01	0.57	− 1.19	− 0.16	0.18	− 0.02	− 0.20	− 0.08	− 0.32^e^
*Trans*-1,2-dichlorocyclohexane	ax,ax-eq,eq	**1.65**	1.19	− 0.88	− 1.20	**− 2.03**	**− 2.02**	− 0.72	**− 1.56**	− 1.02	− 0.33	− 0.11	− 0.36	− 0.88	− 0.96	− 0.60^f^
*Trans*-1,4-difluoro-cyclohexane	ax,ax-eq,eq	0.78	0.67	− 0.44	− 0.94	− 0.42	**− 2.59**	**− 1.65**	− 0.24	− 1.60	− 0.69	− 0.40	− 0.89	− 1.03	− 0.90	− 1.25^e^
*Trans*-1,4-dichlorocyclohexane	ax,ax-eq,eq	**2.41**	**1.64**	0.47	− 0.01	− 0.12	**− 2.01**	0.15	0.30	− 0.04	0.85	1.14	0.52	− 0.32	− 0.31	0.09^d^
**Νο. wrong conformers**		6	7	3	1	1	5	3	3	1	1	3	2	2		
**RMSD**		1.79	2.04	0.58	0.49	0.65	1.23	0.67	0.71	0.85	0.96	1.04	0.73	0.14		

*n.d.* not determined

^a^Δ*H*, gas phase

^b^Δ*H*, solution, low temp. NMR

^c^Δ*G*, solution, low temp. NMR

^d^Δ*G*, gas phase

^e^CCSD(T)//MP2/6-311G(2df,p)

^f^QCISD/6-311+G(2df,p)//MP2/6-311G*

^e,f^Previous reported highest level theory

^g^In the low energy equatorial or axial conformer phenyl ring C1=C2 bond is eclipsing cyclohexane C1-H bond

^h^Axial and equatorial conformer have an eclipsed orientation as regards rotation around the C-O bond

**Table 4:** Relative conformational energies of some oxygen containing compounds (kcal mol^−1^)

Compound	Description	MM+	MMX	UFF	DREIDING	MM3-96	MMFF94	MM3-00	MM4-08	HF/cc-pVDZ	HF/cc-pVTZ	HF/CBS	B3LYP 6-31G(d,p)	MP2/cc-pVDZ	DLPNO-CCSD(T)	Expt. or high level theory^g^
Formic acid	*E-Z*	3.93	**2.26**	− 1.44	− 1.18	3.98	4.90	**2.46**	3.90	5.69	4.97	4.73	4.74	4.55	4.22	3.90^a^
Methyl formate	*E-Z*	3.69	**3.74**	*− *3.26	**− 1.44**	4.94	5.28	3.65	4.77	5.96	5.42	5.25	5.26	5.39	5.22	4.75^b^
Methyl acetate	*E-Z*	3.98	5.15	**1.58**	0.36	7.80	8.27	8.31	7.25	8.90	8.27	8.06	7.70	7.53	7.27	8.5^b^
Propanal	*skew-eclipsed*	0.90	0.95	**− 1.55**	− 1.03	1.18	0.53	1.06	1.28	1.47	0.97	0.80	0.81	1.15	1.04	0.95^a^
2-Butanone	*skew-eclipsed*	**1.59**	1.60	− 1.05	− 0.60	1.47	0.83	1.39	1.43	**2.10**	**1.66**	**1.51**	1.34	1.42	1.34	2.0^a^
Vinyl alcohol	*s-trans*,*s-cis*	**1.81**	1.01	**− 2.28**	**− 1.96**	**1.77**	1.43	**1.58**	1.25	**2.11**	**1.58**	1.41	1.46	**1.68**	1.30	–
Methyl vinyl ether	*g, s-cis*	1.07	3.05	*− *7.53	*− *5.95	**2.44**	**2.52**	3.73	**2.15**	**1.95**	**1.62**	**1.51**	**1.99**	**2.87**	**2.45**	1.15^b^
Glycolic acid	*sk-ecl*	**2.76**	0.90	0.68	0.52	**2.40**	0.80	**1.81**	1.04	0.32	0.27	0.25	0.12	0.49	0.41	4.2^b^
Methyl glycolate	*sk-ecl*	4.66	0.86	0.73	0.57	**2.09**	**2.44**	**1.61**	1.05	0.47	0.21	0.12	− 0.02	0.31	0.20	–
2-Methylcyclo hexanone	*ax-eq*	**2.31**	**2.32**	− 0.35	− 0.26	**2.19**	1.32	**2.07**	**2.09**	**2.75**	**2.27**	**2.12**	**1.92**	**1.84**	**1.74**	1.58^c^
Cyclodecanone	*1keto-3keto*	**2.82**	**2.82**	1.25	**2.09**	**2.57**	3.11	**2.49**	3.13	3.83	3.40	3.26	3.09	3.47	3.21	–
	*2keto-3keto*	4.86	4.87	5.51	5.22	4.34	4.37	4.32	4.09	5.80	5.05	4.81	3.81	**5.04**	**4.39**	–
Ethanol	*g-a*	0.60	0.50	0.46	0.50	0.42	0.18	0.42	0.29	0.05	0.24	0.30	0.07	0.06	0.16	0.129^b^
Ethyl methyl ether	*g-a*	**1.74**	**1.55**	**1.77**	1.49	1.48	**1.50**	**1.50**	1.21	**1.78**	**1.85**	**1.88**	**1.53**	1.40	1.31	1.5^b^
1-Propanol	*ga-aa*^h^	0.31	0.31	0.39	0.15	0.30	0.29	0.36	− 0.18	− 0.19	0.06	0.14	0.03	− 0.26	− 0.10	− 0.3^b^
2-Propanol	*g-a*^i^	− 0.62	− 0.53	− 0.80	− 0.42	− 0.65	− 0.17	− 0.67	− 0.40	− 0.06	− 0.29	− 0.37	− 0.22	− 0.23	− 0.27	− 0.28^a^
2-Propen-1-ol	*sk,a - sk,g*+	0.14	0.41	− 0.38	− 0.60	1.02	1.13	0.40	0.10	1.39	1.06	0.95	1.40	1.38	1.14	–
2-Propen-1-ol	*ecl,a - sk, g*+	0.54	1.40	1.39	0.53	1.20	0.96	0.45	1.39	0.64	0.73	0.75	1.22	0.82	0.64	–
1,2-Ethanediol	*a,a,a - g-,g*+*,a*	0.76	0.06	− 0.69	− 0.61	**2.81**	**2.91**	**2.04**	**2.27**	**2.08**	**1.69**	**1.57**	**2.64**	**2.84**	**2.57**	–
1-MeO-cis-2,6-diMecyclohexane	*ecl-anti*	0.84	0.94	*− *5.80	− 0.57	1.38	0.77	− 1.36	− 1.32	**− 1.81**	**− 1.96**	**− 2.01**	− 1.28	− 0.73	− 0.74	–
C-Et glycoside, O-C_1_-C_exo_-C	*g-a*	− 1.41	− 1.39	**− 2.07**	**− 1.82**	**− 1.50**	**− 2.01**	− 1.47	− 1.48	**− 1.68**	**− 1.72**	**− 1.73**	− 1.45	**− 1.63**	**− 1.54**	~ − 1.6^d^
2-Methyl tetrahydropyran	*ax-eq*	**2.64**	**2.43**	3.99	**1.90**	**2.88**	**2.22**	**2.86**	**2.65**	3.44	3.55	3.59	3.35	**2.95**	**2.78**	2.86^e^
3-Methyl tetrahydropyran	*ax-eq*	1.26	1.27	1.11	0.87	1.27	1.01	1.28	0.68	**1.51**	**1.72**	**1.79**	**1.70**	1.09	1.15	1.50^e^
4-Methyl tetrahydropyran	*ax-eq*	**1.75**	**1.74**	**1.72**	1.33	**1.76**	**1.33**	**1.76**	**1.76**	**2.63**	**2.65**	**2.65**	**2.54**	**2.08**	**1.93**	1.95^e^
2,5-Dimethyl-1,3-dioxane	(2*eq*,5*ax*)–(2eq,5*eq*)	0.71	0.64	0.61	0.39	0.79	0.51	0.78	0.82	0.60	1.05	1.20	1.21	0.49	0.70	0.92^f^
Dimethoxy methane	*ag-g*^*−*^*g*^*−*^	**1.90**	0.46	− 0.81	− 0.16	**2.25**	**2.11**	**2.13**	**2.22**	**2.43**	**1.97**	**1.81**	**2.53**	**2.73**	**2.58**	–
	*aa -g*^*−*^*g*^*−*^	3.90	0.91	− 1.40	− 0.46	4.48	4.58	4.16	5.03	5.59	4.56	4.22	5.86	5.99	5.71	–
	*g eclipsed*^j^* -g*^*−*^*g*^*−*^	**2.26**	**1.67**	0.85	**2.57**	3.17	4.28	3.01	4.05	4.11	3.73	3.61	3.74	3.86	3.54	–
Acetaldehyde dimethyl acetal	*g eclipsed*^j^*-g*^*−*^*g*^*−*^	0.45	0.27	*− *3.27	0.62	0.46	**2.29**	0.26	**2.19**	0.93	0.59	0.48	0.89	1.25	1.17	–
2-Methoxy tetrahydropyran	*ax-eq*	− 1.08	− 0.14	1.41	0.17	− 0.82	**− 1.81**	− 0.76	− 1.26	− 1.28	− 0.65	− 0.45	− 0.78	− 1.40	− 1.21	− 1.27^a^
2-F-tetrahydropyran	*ax-eq*	− 0.51	− 0.75	1.03	0.66	n.d.	**− 2.39**	− 0.51	− 0.92	**− 2.73**	**− 2.02**	**− 1.79**	**− 2.91**	**− 2.44**	**− 2.43**	− 2.45^g^ CCSD(T)
*α,α*-Trehalose	*tgctgr*180180^k^- *gtxgtx*16080-	–^l^	− 1.00	− 1.37	− 0.87	–^l^	0.98	4.18	**2.15**	5.14	7.37	8.09	5.77	6.41	6.96	–
*α,α*-Trehalose	*gtxgtx*6060- *gtxgtx*16080-	–^l^	*− *3.75	*− *17.58	*−* 8.58	–^l^	− 1.24	**− 2.14**	**− 4.74**	− 0.76	− 0.40	− 0.28	− 0.06	0.04	0.04	–
	**Νο. wrong conformers**	4	4	17	16	4	3	2	1	1	2	2	3	1		
	**RMSD**	1.38	2.04	4.93	3.59	0.74	1.26	1.03	1.28	0.74	0.56	0.62	0.40	0.24		

*n.d.* not determined

^a^Δ*G*°, gas phase

^b^Δ*H*°, gas phase

^c^Δ*H*°, solution, low temp. NMR

^d^Δ*G*, solution, NMR, coupling constants ^3^*J*[^1^H-^1^H]

^e^Δ*G*, solution, low temp. NMR

^f^Δ*H*, solution

^g^CCSD(T)/au-cc-pVDZ//MP2/6-311G(2df,2pd) from Ref. [38] (previous reported highest level theory)

^h^The first letter refers to conformer description around C2-C3 bond, the second to conformer description around C-O bond

^i^Rotation around H-C-O-H dihedral Angle

^j^Conformers included by the C1-O2-C3-O4-C5 segment; aa: the two torsion angles between Cl and O4 and C5 and O2 are ca. 180°, g-g-: the torsion angle between Cl and O4 and between C5 and O2 is rotated in a counterclockwise direction by ca. − 60°, ag: one torsion angle between vicinal C and O is ca. 180° and the other is ca + 60 or − 60°, g,eclipsed: one torsion angle between vicinal C and O is eclipsed

^k^gtxgtx16080: dihedral angles C5-O5-C1-O1 and O5-C1-O1-C1′ are 160° and 80°, respectively; gt corresponds to a conformer in which the primary hydroxy group is in trans position relative to Ο-5 and gauche relative to C4 and x indicates a trans conformer for H-C2-O2-H dihedral angle [107]

^l^MM3 and MM+ force fields did not calculate gtxgtx16080 and tgctgr180180 as stable conformations

**Table 5:** Relative conformational energies of some nitrogen containing compounds (kcal mol^−1^)

Compound	Description	MM+	MMX	UFF	DREIDING	MM3-96	MMFF94	MM3-00	MM4-08	HF/cc-pVDZ	HF/cc-pVTZ	HF/CBS	B3LYP 6-31G(d,p)	MP2/cc-pVDZ	DLPNO-CCSD(T)	Expt. or high level theory^b,e^
Ethylamine	*g-a*^f^	− 0.13	− 0.02	− 0.65	− 0.43	− 0.10	− 0.44	− 0.13	− 0.21	0.08	− 0.11	− 0.16	0.07	0.00	− 0.09	0.306, 0.20^a^
Methylethylamine	*Gg-Tg*^g^	1.17	0.99	2.36	1.11	1.14	**1.86**	1.16	0.48	1.20	1.20	1.20	1.41	1.32	0.73	–
	*Gt-Tg*	1.04	1.04	1.41	0.97	1.16	**1.61**	1.17	1.03	**1.59**	**1.66**	**1.68**	0.96	0.78	1.26	–
1-Propylamine	*Gt-Tt*^l^	0.67	0.69	0.50	0.21	0.48	0.39	0.48	0.39	0.06	0.33	0.42	0.36	− 0.08	0.08	–
2-Propylamine	*g-a*	0.14	0.03	0.35	0.14	0.22	0.45	0.26	0.32	0.16	0.39	0.46	0.34	0.39	0.46	0.45^a^; 0486^b^
Pyrrolidine	N-H (*ax*-*eq*), *E*(2)^h^	0.30	0.07	− 0.27	0.17	0.39	0.89	0.34	0.60	0.62	0.71	0.73	0.21	0.13	0.05	0.21^b^
Hexahydropyrimidine	NH,NH (*ax,eq-ax,ax*)	1.10	**2.71**	− 0.70	− 0.44	1.30	**1.99**	− 1.12	**− 2.50**	0.22	0.25	0.26	0.03	0.03	− 0.05	–
3-OH-hexahydro-pyrimidine	N-H,N-H,O-H (*ax,ax,exo-ax,eq,endo*)	− 0.41	− 1.05	− 0.84	− 0.52	3.50	4.00	2.12	4.18	0.69	0.39	0.29	0.97	1.37	1.17	–
Piperidine	*ax-eq*	0.31	0.09	0.77	0.58	0.29	0.90	0.34	0.60	0.91	0.97	0.99	0.76	0.85	0.78	0.53^a^
*N*-methylpiperidine	*ax-eq*	**2.53**	**2.10**	3.73	**1.69**	**2.32**	3.28	**2.38**	3.52	3.98	4.08	4.11	3.94	3.61	3.45	3.15^c^
2-Methylpiperidine	*ax-eq* (*N*-Heq)	**2.12**	**1.95**	3.14	**1.56**	**2.35**	**2.38**	**2.39**	**2.47**	3.17	3.32	3.37	3.23	**2.91**	**2.76**	2.50^d^
3-Methylpiperidine	*ax-eq* (*N*-Heq)	**1.63**	**1.65**	1.27	0.93	1.48	1.09	1.48	2.02	1.59	1.83	1.90	1.79	1.10	1.23	1.60^d^
4-Methylpiperidine	*ax-eq* (*N*-Heq)	**1.74**	**1.73**	**1.82**	1.32	**1.74**	1.38	**1.75**	1.49	**2.62**	**2.66**	**2.67**	**2.51**	**2.04**	**1.90**	1.90^d^
*N*,2-dimethylpiperidine	*N*-Me,C-Me (*eq,eq*-*eq,ax*)	1.65	1.20	*− *2.03	**0.35**	1.56	1.57	**− 1.62**	− 0.56	**− 1.61**	**− 1.84**	**− 1.92**	**− 2.08**	**− 2.19**	**− 2.08**	1.80^d^
*N*,3-dimethylpiperidine	*N*-Me,C-Me (*eq,eq*-*eq,ax*)	1.59	1.62	− 1.22	− 0.89	1.45	1.06	− 1.46	**− 2.32**	**− 1.54**	**− 1.80**	**− 1.88**	**− 1.83**	− 1.01	− 1.16	1.60^d^
*N*,4-dimethylpiperidine	*N*-Me,C-Me (*eq,eq*-*eq,ax*)	1.73	1.71	**− 1.83**	− 1.29	1.71	1.37	**− 1.73**	− 1.49	**− 2.56**	**− 2.60**	**− 2.61**	**− 2.43**	**− 1.89**	**− 1.80**	1.80^d^
2-(1-Ad)-*N*-Me-piperidine	C-Ad,*N*-Me (*eq,eq*-*eq,ax*)	1.11	**1.74**	**3.62**	4.83	1.30	1.32	1.24	**− 1.66**	**2.48**	**2.18**	**2.08**	1.33	1.27	1.23	1.40^d^
2-(2-Ad)-*N*-Me-piperidine	C-Ad,*N*-Me (*ax,ax-eq,ax*)	0.96	0.63	**− 1.24**	**− 1.10**	0.09	− 0.08	0.08	*− *2.92	0.31	0.50	0.56	0.50	0.64	0.74	1.20^d^
Ethanediamine	*gGg′-tTt*^i^	− 1.17	− 0.62	0.39	0.18	− 1.13	− 1.19	− 1.06	− 1.45	− 0.23	− 0.35	− 0.39	− 1.44	− 1.19	− 1.17	–
Propanediamine	*gGGg′-tTTt*^i^	0.65	1.52	0.79	0.58	− 0.64	1.23	0.00	0.45	− 0.68	− 0.26	− 0.12	− 1.10	**− 1.82**	**− 1.59**	–
Butanediamine	*gGGGg′-tTTTt*^i^	**3.12**	4.01	4.09	**3.25**	**1.71**	− 0.07	**2.56**	**2.42**	**1.74**	**2.58**	**2.86**	0.64	− 0.26	0.04	–
3β-Aminotropane	1c-1a^j^	0.49	0.38	7.49	1.25	1.04	0.32	1.15	0.38	0.41	0.17	0.09	0.0	0.53	0.39	–
Ethyldimethyl ammonium	*g-a*	0.62	− 0.17	0.42	0.51	0.90	0.78	0.62	0.72	1.01	1.09	1.11	0.97	0.86	0.84	–
*N*-Me-piperidinium	*N*-Me (*ax*-*eq*)	**2.24**	0.74	**1.83**	**2.07**	**1.95**	**2.01**	**1.66**	**− 2.94**	**2.71**	**2.81**	**2.84**	**2.47**	**2.16**	**2.11**	2.10^d^
3β-Aminotropan-dication	3c-3a^j^	− 0.11	0.24	**2.77**	**2.00**	**2.25**	1.31	**2.59**	1.06	**2.24**	**2.24**	**2.24**	**1.86**	**2.21**	**2.14**	–
3β-Aminotropane monocation	2a-2b^j^	*− *7.21	*− *3.85	*− *12.65	*− *10.82	5.96	0.69	9.05	*− *15.19	**1.83**	0.52	0.09	4.45	4.80	3.91	–
Ethanediamine monocation	*Gg-Tg*^i^	0.28	*− *2.30	0.41	0.16	*− *5.17	*− *14.26	*− *11.16	*− *10.59	*−* 7.86	*− *6.80	*− *6.46	*− *10.41	*− *10.93	*− *10.14	–
*N*-methylacetamide	*E*-*Z*	**2.18**	0.67	0.45	0.74	**2.60**	**2.18**	**2.94**	**2.69**	**2.51**	**2.48**	**2.47**	**1.91**	**2.07**	**2.11**	2.23^d^
*N*-methylformamide	*E*-*Z*	0.83	− 0.30	− 0.97	− 0.52	**1.52**	1.28	**1.84**	**2.11**	0.94	0.97	0.98	0.86	1.12	1.08	1.30^d^
Formamidine	*cis–trans* (H-N=C-N)	− 0.01	0.11	1.43	0.51	n.d.	**2.27**	− 0.89	− 0.44	1.23	**1.80**	**1.98**	**2.12**	**1.71**	**1.72**	–
*N*-Me formamidine	*cis–trans* (N-C=N-C)	0.45	0.35	**1.66**	0.80	n.d.	**2.12**	3.80	**1.76**	**2.30**	**2.39**	**2.42**	**2.21**	1.34	1.22	–
*N*-acetylglycine-*N*-methylamide	*aa-g*^+^*g*^*−*^ (or C5-C7)^k^	3.99	1.20	4.34	**2.89**	4.03	1.31	**2.91**	3.54	− 0.39	− 0.82	− 0.97	0.89	**1.68**	**1.55**	–
*N*-acetylalanine-*N*-methylamide	*aa-g*^+^*g*^*−*^ (C5-C7eq)^k^	4.35	**1.95**	0.04	0.46	3.27	**1.53**	**2.03**	4.80	0.10	− 0.58	− 0.80	**1.71**	**1.93**	**1.79**	–
	*g*^*−*^*g*^+^*-g*^+^*g*^*−*^ (C7ax-C7eq)	**1.78**	1.13	4.13	**1.89**	1.46	**2.45**	1.08	**1.84**	3.14	**2.95**	**2.88**	**2.51**	**2.44**	**2.35**	–
NAPA	C5-C7eq,g+^k^	3.59	− 0.11	*− *3.26	**− 2.20**	0.44	0.48	− 0.26	1.45	− 0.53	**− 1.61**	**− 1.96**	0.0	0.32	0.07	0.56^e^
NAPA	C7eq,*g*^*−*^*-*C7eq,*g*+^k^	− 0.82	**− 1.52**	*− *3.16	**− 2.42**	0.92	0.78	0.04	**− 2.38**	− 0.04	− 0.65	− 0.85	0.25	1.01	0.58	0.57^e^
NAPA	C7eq,*a*-C7eq,*g*+^k^	0.17	− 0.77	**− 1.81**	− 1.16	**2.97**	1.01	**1.74**	0.10	− 0.29	− 0.70	− 0.83	0.40	1.28	0.99	0.64^e^
	**Νο. wrong conformers**	11	12	11	11	4	7	2	7	6	6	6	4	4		
	**RMSD**	2.96	2.43	3.79	3.29	1.60	1.48	1.29	3.58	0.91	1.25	1.37	0.40	0.25		

*n.d.* not determined

^a^Δ*H*, gas phase

^b^CCSD(T)/aug-cc-pVTZ//MP2/aug-cc-pVTZ

^c^Δ*G*, gas phase

^d^Δ*G*, solution, DNMR

^e^CASSCF/MS-CASPT2//B3LYP/6-31 + G**

^b,e^Previous reported highest level theory

^f^Refers to dihedral lp-N-C-C

^g^The first chapter letter refers to C-N-C-C dihedral and the second letter refers to lp-N-C-C dihedral

^h^Carbon C-2 is outside of the plane of the other atoms

^i,j,k^See Figs. 17, 18, and 19

**Table 6:** Conformational energies of some compounds containing sulfur and phosphorus (kcal mol^−1^)

Compound	Description	MM+	MMX	UFF	DREIDING	MM3-96	MMFF94	MM3-00	MM4-08	HF/cc-pVDZ	HF/cc-pVTZ	HF/CBS	B3LYP 6-31G(d,p)	MP2/cc-pVDZ	DLPNO-CCSD(T)	Expt.
Ethanethiol	*g-a*^e^	− 0.29	− 0.06	0.14	0.23	− 0.29	− 0.68	− 0.29	− 0.53	− 0.45	− 0.33	− 0.29	− 0.61	− 0.51	− 0.42	− 0.41^a^
2-Propanethiol	*g-a*^e^	0.27	0.06	− 0.17	− 0.16	0.28	0.69	0.54	0.30	0.17	− 0.03	− 0.09	0.13	0.04	− 0.05	0.06^a^
Methylethylsulfide	*g-a*^e^	0.38	0.38	1.09	1.12	0.13	− 0.32	0.13	0.07	0.26	0.40	0.44	0.07	− 0.01	0.00	− 0.45^b^
Methylethylsulfone	*g-a*^f^	0.79	− 0.14	− 0.70	0.15	0.43	1.42	0.43	0.11	0.48	0.52	0.53	0.09	0.21	0.22	–
2-Methylthiane	*ax-eq*	1.21	1.21	**2.57**	**1.54**	1.04	0.11	0.99	1.04	**1.79**	**2.03**	**2.10**	**1.89**	**1.55**	**1.54**	1.42^c^
3-Methylthiane	*ax-eq*	1.44	1.44	**1.81**	1.02	**1.61**	0.69	**1.60**	0.86	**1.77**	**2.03**	**2.11**	**1.92**	1.28	1.33	1.40^c^
4-Methylthiane	*ax-eq*	**1.81**	**1.81**	**2.31**	1.39	**1.82**	1.31	**1.82**	1.47	**2.33**	**2.42**	**2.45**	**2.30**	**1.76**	**1.70**	1.80^c^
S-methylthianium	*ax-eq*	1.12	1.39	7.06	**2.95**	**1.52**	4.98	1.07	1.25	0.93	1.29	1.41	0.83	0.77	0.76	0.30^c^
Thiacyclohexane	*twb-ch*	3.80	4.15	7.57	7.19	5.07	4.08	5.07	5.30	5.45	5.34	5.31	5.14	5.01	4.69	4.02^a^
Sulfolane	C_s_-C_2_^g^	0.39	**1.55**	12.16	**1.99**	**2.39**	5.09	**1.63**	**3.44**	**1.64**	1.44	1.38	**1.94**	1.43	1.21	–
	**Νο. wrong conformers**	1	2	2	1	1	1	1	1	1	0	0	1	1		
	**RMSD**	0.43	0.37	2.40	1.20	0.37	1.60	0.35	0.79	0.39	0.48	0.87	0.36	0.12		
Ethylphosphine	*g-a*^h^	0.59	n.d.	− 0.001	0.02	0.57	− 0.08	0.55	0.62	0.37	0.28	0.25	0.55	0.55	0.49	0.57^a^
Ethyldimethylphosphine	*g-a*^h^	− 0.94	− 1.01	**− 2.89**	*− *5.44	− 0.41	**− 2.16**	− 0.41	− 0.75	− 1.23	− 1.31	− 1.34	− 1.07	− 0.93	− 0.88	− 0.38^a^
Tri-OMe phosphate	*ggg-ggt*^i^	− 0.31	n.d.	0.18	0.21	**− 2.25**	n.d.	n.d.	n.d.	0.68	1.26	1.45	0.84	0.64	0.83	
P-Me-phosphorinane	*ax-eq*	0.11	0.30	**2.54**	5.33	− 0.80	1.43	− 0.81	− 0.12	0.62	0.87	0.96	0.72	0.57	0.51	0.70^d^
	**Νο. wrong conformers**	1	0	1	0	2	1	1	1	0	0	0	0	0		
	**RMSD**	0.24	0.17	1.67	3.84	0.80	0.97	0.81	0.38	0.22	0.35	0.39	0.17	0.06		

*n.d.* not determined

^a^ΔH, gas phase

^b^ΔG, gas phase

^c^ΔG, solution, low temp. NMR

^d^ΔH, solution, low temp. NMR

^e^Rotation around C-S bond (dihedral C-C-S-C or C-C-S-H)

^f^Rotation around CH_2_-SO_2_ bond

^g^C2: half-chair conformation, C_s_: envelope conformation

^h^Rotation around dihedral C-C-P-lp

^i^Rotation around P-O bond

**Table 7:** Relative conformational energies of some conjugated compounds (kcal mol^−1^)

Compounds	Description	MM+	MMX	UFF	DREIDING	MM3-96	MMFF94	MM3-00	MM4-08	HF/cc-pVDZ	HF/cc-pVTZ	HF/CBS	B3LYP/6-31G(d,p)	MP2/cc-pVDZ	DLPNO-CCSD(T)	Expt. or high level theory^b,c^
1,3-Butadiene	*s,cis-s,trans*	**2.35**	1.18	3.35	**2.17**	**1.94**	**2.47**	**1.86**	**2.53**	4.08	4.29	4.36	3.96	3.74	3.50	2.94^a^, 3.01^b^
Propenal (acrolein)	*cis–trans*	**1.71**	**2.43**	**1.71**	0.62	**1.76**	**2.04**	**1.52**	**1.84**	**1.59**	**2.27**	**2.50**	**2.15**	**1.99**	**2.01**	2.20^a^, 2.06^b^
2-Methylpropenal (methacrolein)	C=C-C=O *cis-trans*	**2.60**	4.28	0.80	0.26	**2.22**	3.08	n.d.	n.d.	**1.62**	**1.96**	**2.08**	**2.11**	**2.19**	**2.09**	3.02^a^, 3.47^b^
Methyl vinyl ketone	C=C-C=O *cis–trans*	0.69	1.32	− 0.92	− 0.60	0.77	0.62	0.78	0.79	− 0.40	0.13	0.31	0.11	0.36	0.52	0.80^a^, 0.61^b^
2-Methyl-1,3-butadiene	*s,cis-s,trans*	1.37	1.01	**2.62**	0.93	**1.63**	**2.46**	**1.64**	3.08	6.36	6.61	6.69	5.69	6.20	5.84	2.65^a^
	**Νο. wrong conformers**	0	0	1	1	0	0	0	0	1	0	0	0	0		
	**RMSD**	2.08	2.62	1.69	2.55	2.01	1.64	2.27	1.47	0.61	0.54	0.59	0.29	0.21		

*n.d.* not determined

^a^Δ*Η*, gas phase

^b^CCSD(T)(FC)/CBS + CCSD(T)(CV)/cc-pwCVQZ + scalar relativistic effects correction + CCSDT(Q)(FC)/cc-pVDZ correction

^c^CCSD(T)/CBS

^b,c^Previous reported highest level theory

**Table 8:** Intramolecular conformational barriers (kcal mol^−1^)

Compounds	Description	MM+	MMX	UFF	DREIDING	MM3-96	MMFF94	MM3-00	MM4-08	HF/cc-pVDZ	HF/cc-pVTZ	HF/CBS	B3LYP 6-31G(d,p)	MP2/cc-pVDZ	DLPNO-CCSD(T)	Expt. or high level theory^b,d^
Ethane	C-C	**2.73**	**2.73**	**2.90**	**2.90**	**2.41**	3.21	**2.41**	**2.60**	3.40	3.19	3.12	**2.74**	3.03	**2.83**	2.88^a^
Propane	C-C	3.04	3.04	3.48	3.37	**2.68**	3.41	**2.68**	**2.81**	3.60	3.44	3.39	3.02	3.30	**3.09**	3.27^a^
Butane	C-C	4.73	4.73	7.70	5.81	4.84	5.21	4.83	4.94	6.65	6.59	6.57	5.79	5.77	**5.48**	5.40^b^
Ethanol	C-C^f^	**2.71**	**2.68**	3.27	3.23	**2.79**	3.40	**2.80**	3.40	3.88	3.67	3.60	3.16	3.56	**3.34**	3.32^a^
Bicyclooctyl-CMe_2_Cl	C-C	7.22	8.14	12.53	10.84	11.04	11.49	11.02	9.35	9.92	11.28	11.73	7.45	10.10	**9.88**	9.8^c^
t-Bu-CMe_2_Cl	C-C	7.04	7.05	12.72	11.14	10.81	11.31	10.84	9.43	9.90	10.84	11.15	8.02	10.25	**10.06**	10.4^c^
1-adamantyl-CMe_2_Cl	C-C	7.35	7.38	13.05	11.35	11.05	11.65	11.08	9.39	10.17	11.69	12.18	7.52	10.74	**10.55**	9.30^c^
ethylamine	C-C^f^	**2.66**	**2.65**	3.30	3.17	2.99	3.72	3.00	3.23	4.30	4.11	4.05	3.33	4.02	**3.74**	3.74^a^
2-(1-adamantyl)-*N*-methylpiperidine	C-C	8.41	**8.30**	10.40	10.01	8.06	11.88	9.63	10.13	9.43	9.39	9.38	6.64	8.71	**8.52**	7.6^c^
Ethanethiol	C-C^g^	3.55	3.56	**2.39**	3.31	3.66	4.05	3.66	3.44	4.15	3.93	3.86	3.59	3.84	3.56	3.77^a^
Acetone	CO-C	0.77	0.78	1.07	1.03	0.74	0.83	0.74	0.76	0.79	0.72	0.70	0.51	0.67	0.57	0.78^a^
Methanol	C-O	0.90	0.76	1.04	**2.66**	0.78	1.23	0.79	1.06	1.10	0.94	0.88	1.16	0.80	0.67	1.07^a^
Dimethylether	C-O	**2.53**	**2.18**	**2.17**	**2.97**	**2.45**	**2.43**	**2.45**	**2.68**	**2.40**	**2.34**	**2.33**	**2.50**	**2.96**	**2.71**	2.63^a^
Methanethiol	C-C^g^	**2.29**	0.03	1.30	**2.38**	1.28	1.38	1.28	1.27	1.43	1.31	1.27	1.25	1.27	1.13	1.27^a^
Dimethylsulfide	C-S	3.03	**1.99**	**2.08**	**2.89**	**2.25**	1.83	**2.25**	**2.02**	1.23	1.25	1.25	1.16	1.11	1.03	2.13^a^
Dimethylsulfone	C-S	**3.14**	**3.21**	1.16	**2.39**	3.22	**2.94**	3.31	3.36	**2.31**	3.97	4.52	**2.63**	**2.03**	**2.47**	3.40^a^
Methyl formate	CO-O (Z → E)	10.05	9.13	*− *3.26	**− 2.91**	13.66	10.12	16.51	13.66	10.62	10.08	9.91	10.99	11.01	10.71	–
Methyl formate	O-CH_3_	1.19	1.32	**2.81**	3.65	1.20	0.79	1.14	1.17	1.04	1.08	1.09	0.62	0.95	1.00	1.19^a^
*Ν*-methylacetamide	CO-N (E → Z)	20.39	17.24	24.63	0.41	15.79	22.16	16.00	21.56	22.02	21.98	21.98	24.12	23.56	22.75	~ 21.0
Methylamine	C-N	**1.70**	**1.68**	**1.97**	**2.96**	1.45	**2.35**	1.45	**1.95**	**2.30**	**2.05**	**1.96**	**1.91**	**1.81**	**1.62**	1.98^a^
Dimethyl phosphine	C-P	**2.93**	–	**2.33**	**2.93**	**1.80**	**2.50**	**1.94**	**1.95**	4.87	4.86	4.85	1.04	1.10	4.21	–
Propene	=C-C	**2.08**	**2.08**	0.47	0.50	**1.74**	**1.96**	**1.74**	**1.81**	**2.36**	**2.32**	**2.31**	**1.95**	**2.09**	**2.02**	1.99^a^
Cyclohexane	Sofa-chair ring inversion	**10.14**	**10.18**	14.74	11.32	10.53	10.31	10.85	11.56	13.23	12.96	12.87	11.84	12.89	12.25	11.3^a^
Cyclohexene	Boat-half chair	6.07	6.10	5.91	5.30	6.67	7.60	6.58	7.27	6.96	6.80	6.74	5.76	5.87	5.85	5.50^d^
*N*-methylpiperidine	Sofa-chair—NMe(eq) ring inversion	11.19	10.55	13.25	11.02	10.70	12.97	9.56	10.70	11.10	10.72	10.60	9.75	10.87	10.15	12.1^a^
*N*-methylspiro [piperidine-2,2′-adamantane]	Boat–chair NMe(eq) ring inversion	16.40	16.73	9.60	8.04	15.97	16.74	16.24	8.12	17.40	16.93	16.78	15.41	16.13	15.52	15.2^e^
*N*-methylpiperidine	Planar N—NMe (eq) nitrogen inversion	8.88	9.51	**− 1.77**	**2.39**	9.04	12.16	9.10	9.51	8.93	9.00	9.02	8.87	10.90	10.42	8.7^a^
*N*-methylpyrrolidine	Planar N—NMe (eq) nitrogen inversion	4.89	5.58	**− 2.64**	− 1.25	**5.23**	8.85	5.63	6.09	6.20	6.34	6.39	7.89	6.20	7.66	7.2^e^
3,3-Dimethyl-*N*-methylpyrrolidine	Planar N—NMe (eq) nitrogen inversion	4.97	5.69	**− 2.77**	− 1.41	**4.85**	**8.68**	**4.89**	6.07	6.26	6.08	6.02	6.23	7.58	7.18	6.7^e^
	**Νο. favoring wrong conformer**	0	0	4	3	0	0	0	0	0	0	0	0	0		
	**RMSD**	1.48	1.63	4.72	5.84	1.73	1.26	1.91	1.71	0.78	0.82	0.91	1.19	0.67		

^a^Δ*H*, gas phase

^b^CCSD(T)/TZ//CISD/DZP

^c^Δ*G*, solution, DNMR

^d^QCISD(T)/6-13G*/MP2/6-31G*

^b,d^Previously reported highest level theory

^e^Δ*G*, solution, NMR

^f^Energy difference from the global minimum, i.e., the *anti* conformation

^g^Energy difference from the global minimum, i.e., the *gauche* conformation

### Hydrocarbons

**Fig. 1:**
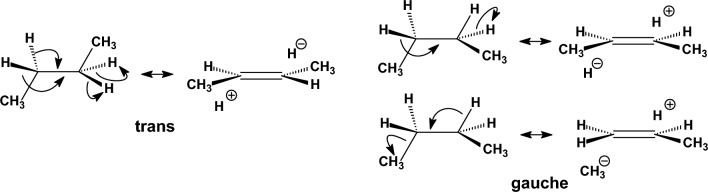
Hyperconjugative interactions in *trans* and *gauche n*-butane. There are four hyperconjugative *σ*_C-Η_ → σ*_C-H_ interactions in *trans* conformation but two *σ*_C-H_ → σ*_C-Η_ and two *σ*_C-Η_ → σ*_C-C_ in gauche conformation

**Fig. 2:**
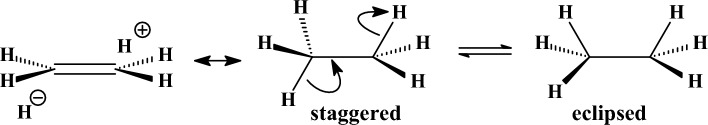
The stabilisation of staggered configuration with respect to eclipsed in ethane comes from the hyperconjugative orbital interactions *σ*_C-H_ → σ*_C-H_

The results of the calculations and experimental data for the studied molecules appear in Table 1. For *n*-butane, the *anti* conformation is stabilized with respect to the *gauche* conformation by experimentally determined energies in the gas phase of 0.69 kcal mol^−1^ [113], 0.67 kcal mol^−1^ [114], 0.71 kcal mol^−1^ [115]. The rotational barriers and conformational energies in the gas phase have been measured [116] and it has been proposed that in the lower-energy *trans* conformer the hyperconjugative orbital interaction between antiparallel C-H bonds, *σ*_C-H_ → σ*_C-Η_ (Fig. 1) contributes to the stabilization of the *anti* conformer [117]. The hyperconjugative phenomenon simply suggests that the Lewis structures for organic molecule representation is an approximation. The importance of hyperconjugative interactions in the conformational analysis of organic molecules was reported in 2001 [118], where it was suggested that the lower energy of staggered compared to the eclipsed ethane results, not from smaller steric repulsions, but from hyperconjugative stabilization (Fig. 2), which is equivalent to the formation of more bonds that lowers the energy. After some rebuttal [119, 120], it has been suggested that both steric effects and hyperconjugative interactions play important roles in stabilizing the staggered conformation in ethane. While steric effects make the dominant contribution [121–123], hyperconjugation interactions contribute about one third of the total torsional barrier in ethane. In butane, the calculated potential energy surfaces and the Natural Bond Orbital (NBO) analysis suggested that the *gauche* conformer is destabilized because of the steric repulsions between the *gauche* methyl groups while hyperconjugative interactions play an important, but not prelevant role for the relative conformational energies [121–123]. All the tested theories calculate the *anti* conformation as the global minimum in accordance to the DLPNO-CCSD(T) theory and experimental values in the gas phase (Table 1) [113–115]. Compared to the DLPNO-CCSD(T) value, the Dreiding, MMFF94, MM+, MMX, MM3-96, MM3-00 force fields have a deviation of ~ + 0.2 kcal mol^−1^, UFF force field and HF theories perform with a deviation of ~ + 0.4 to 0.5 kcal mol^−1^ and B3LYP functional with a deviation of + 0.3 kcal mol^−1^. The MM4-08 force field (+ 0.08 kcal mol^−1^) and MP2 theory (+ 0.03 kcal mol^−1^) have the smallest deviation.

**Fig. 3:**
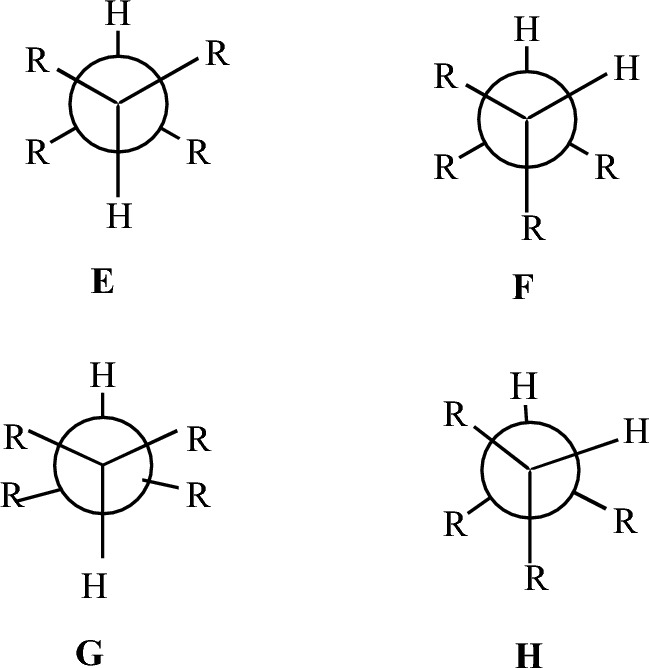
1,1,2,2-Tetrasubstituted ethane conformations. The opening up of bond angles R-C-R caused by steric crowding of methyl groups does not lead to additional unfavourable interactions in *gauche* conformation (F → H) but it causes stereochemical tension in *anti* conformation (E → G)

According to ^13^C-NMR at very low temperatures in 2,3-dimethylbutane the preference for anti conformation, like **E** (Fig. 3) is small compared to the *n*-butane, despite the common observation that the *anti* conformation has only two *gauche* interactions versus three in the *gauche* conformation **F** [124, 125]. This is also confirmed by the DLPNO-CCSD(T) calculations (Table 1). The increase in *gauche* conformation population can be stabilized because steric forces between vicinal methyl groups are reduced through opening up of the Me-C-Me bond angles and steric interactions may be further eased by rotation about the central bond resulted in structure **H** [126]. In contrast, in *anti* conformation **E** there is no option for steric strain relief because opening up of the Me-C-Me bond angles forces the vicinal methyl groups together, as shown in structure **G** [126]. Compared to DLPNO-CCSD(T) theory, the Dreiding, UFF and MMFF94 force fields calculate erroneously the *anti* conformation as the global minimum with an energy deviation in the range of 0.28–1.20 kcal mol^−1^ while HF theories provide energy deviations in the range of 0.10–0.20 kcal mol^−1^. In contrast, the Allinger force fields, B3LYP and MP2 calculate accurately this small conformational energy difference.

**Fig. 4:**
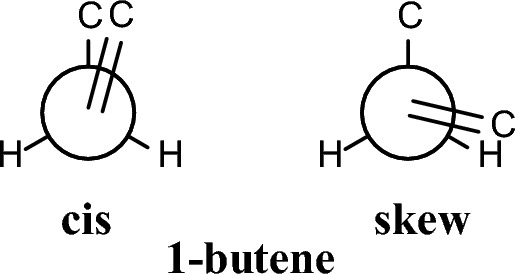
Conformations of 1-butene

In 1-butene (Fig. 4), the *skew* conformation has lower energy than the *cis* conformation, in good accord with the experimental data [127] while DPLNO-CCSD(T) theory calculates the *cis* with a slight lower energy (0.01 kcal mol^−1^) relative to the *skew* conformation (Table 1). Compared to the DPLNO-CCSD(T) calculations, all theories [128], except UFF, stabilize or overstabilize (> 2 kcal mol^−1^ with the Dreiding force field) the *skew* conformation as the global energy minimum. In this case MP2 performed the best. The UFF force field overstabilizes the *cis* conformation by > 3 kcal mol^−1^.

**Fig. 5:**
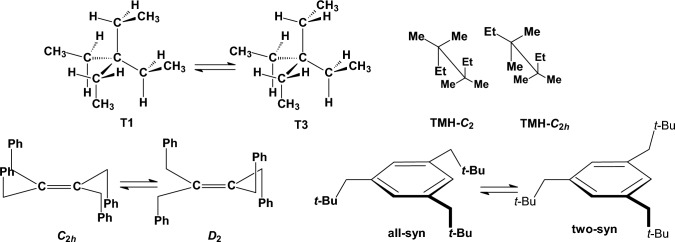
Low energy conformations for tetraethylmethane, tetramethylhexane, tetrabenzylethene and tri-(neopentyl)benzene

Dispersion (attractive van der Waals) forces act at distances longer than the sum of van der Waals radii [129, 130]. We evaluated the ability of the tested methods to calculate the contribution of dispersion interactions in conformational preferences by studying a few relevant molecules, e.g., the 1,2-diphenylethane, tetraethylmethane, tetramethylhexane, tri-neopentyl-benzene and tetra-benzyl-ethene (Fig. 5).

In 1,2-diphenylethane the *gauche* conformations is stabilized by *π–π* interactions compared to the steric relief in the anti conformation. For several decades the results were non-conclusive [131–134] and an experimental energy difference of 1.19 kcal mol^−1^ [134] or 0.57 kcal mol^−1^ [132] in favor of the *anti* conformation was suggested. However, a recent computational chemistry and spectroscopic investigation showed that 1,2-diphenylethane exists as a mixture of *gauche* and *anti* conformations, with the *gauche* being the global minimum [135]. Our DPLNO-CCSD(T) calculations suggest the stabilization of the *gauche* conformation compared to the *anti* conformation albeit only by 0.32 kcal mol^−1^. Compared to the DPLNO-CCSD(T) calculations all the Allinger force fields, the HF theories and the conventional B3LYP functional stabilize or overstabilize the *anti* conformation while low-order post-HF (MP2) approaches strongly favor the *gauche* conformation [135]. However, inclusion of semiempirical dispersion effects in density functionals or coupled cluster post-HF models agree in forecasting the simultaneous presence of both conformers in the gas phase with a slightly larger stability (0.32 kcal mol^−1^) of the gauche conformation. Surprisingly, Dreiding and UFF predict *gauche* conformer as the global minimum for 1,2-diphenylethane with Dreiding performing with an error > 2 kcal mol^−1^.

In tetraethylmethane [136] the T1 conformation is lower in energy than the T3 conformation, according to our DPLNO-CCSD(T) calculations, in good agreement with the dynamic NMR data [136]. In T1 conformation compared to T3 conformation the two methyl groups are in a *syn* position (as shown in the upper and right part of the T1 conformation) where dispersion forces act stabilizing more T1 compared to the T3 conformation. Except for UFF, which calculates both T1 and T3 conformations with equal stability, all the other theories calculate the right global minimum (T1 conformation) for tetraethylmethane. The DPLNO-CCSD(T) calculations show that in tetramethylhexane [137] the *C*_2*h*_ conformation has almost equal energy-slightly lower—compared to *C*_2_ conformation. In the *C*_2*h*_ conformation the two ethyl groups are in a *syn* instead of an *anti* orientation, respectively, and attractive London dispersion (LD) forces antagonize Pauli repulsion (steric hindrance) forces leading to equal energies of *C*_2*h*_ and *C*_2_ conformations according to the DPLNO-CCSD(T) calculations, in contrast to dynamic NMR in solution where *C*_2*h*_ is prevailed in the ~ 60:40 mixture with *C*_2_ (Δ*G* = 0.22 kcal mol^−1^). UFF, Dreiding, MMFF94, MM3-96 calculate clearly C_2*h*_ as the global minimum while MM3-00 and MM4-08 predicted clearly the C_2_ as the global minimum for tetramethylhexane. MM+, MMX, HF theorries, B3LYP and MP2 theories calculate correctly that *C*_2*h*_ and *C*_2_ conformations are equal in energy.

In tetrabenzylethene [103] or 1,3,5-trineopentyl benzene, the benzyl or t-butylmethyl substituents form *π–π* or alkyl–alkyl dispersive interactions when they are in a *syn* orientation.

In tetrabenzylethene, in the *D*_2_ conformation, which is also observed in the solid state for the benzene dimer, the phenyl groups, each linked through a methylene to the same unsaturated carbon, are in *anti* orientation while in *C*_2*h*_ all benzene rings are in *syn* orientation. In *C*_2*h*_ conformation the phenyl groups are in *syn* orientation and dispersion attraction antagonize Pauli repulsion. In tetrabenzylethene the *D*_2_ conforation is clearly more stable by 4.5 kcal mol^−1^ than *C*_2*h*_ conformation according to our DPLNO-CCSD(T) calculations suggesting that the repulsive interactions prevail. Compared to the DPLNO-CCSD(T) calculation the MM+ and UFF calculate the wrong global minimum. Dreiding, MMX, MMFF94 and particularly the MM3-96, MM3-00, MM4-08 force fields underestimate the energy difference, and the HF theories overestimate the energy difference, with Allinger force fields and HF theories performing with deviation > 3 kcal mol^−1^. The B3LYP (− 1.20 kcal mol^−1^) and MP2 (− 0.67 kcal mol^−1^) perform the best with this model molecule with the latter having a smaller deviation.

In 2,4,6-tribromo-1,3,5-trineopentyl benzene the *all-syn* conformation is more stable than the *two-syn*, as shown by the DPLNO-CCSD(T) calculations (1.45 kcal mol^−1^) and also as observed experimentally by dynamic NMR (1.05 kcal mol^−1^) [138]. This is due to the dispersive forces between the *all-syn t*-butyl groups which seem to prevail over the repulsive forces (Fig. 5). All theories calculate correctly the *all-syn* conformation as the global minimum. Compared to the DPLNO-CCSD(T) calculation, the UFF and Dreiding calculate too high conformational energies (> + 3 kcal mol^−1^) with B3LYP (+ 1.17 kcal mol^−1^) MMFF94 and MM3-96 force fields (~ + 0.8 kcal mol^−1^) and HF theories (~ − 0.7 kcal mol^−1^) having the next larger errors. MM3-00 and MM4-08 force fields and MP2 theory perform with the smallest deviation (~ 0.1 kcal mol^−1^).

**Fig. 6:**
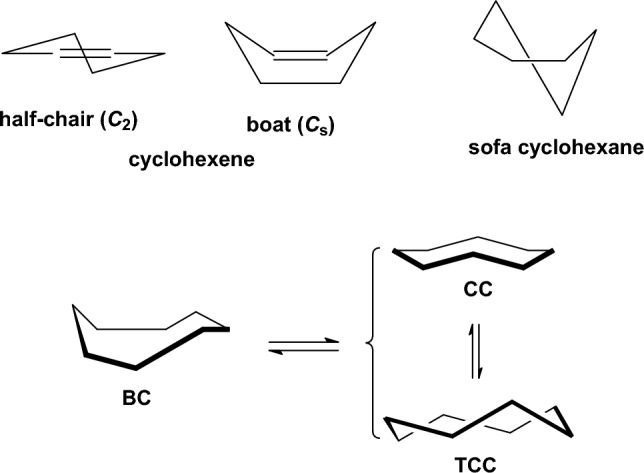
Conformers of cyclohexane, cyclohexene and cyclooctane

For cyclohexane or cyclohexene and cyclooctane it has been shown experimentally in the gas phase or with dynamic NMR in solution, respectively, that the *chair* cyclohexane is more stable over *twist-boat* [112] by 5.5 kcal mol^−1^ [139], the *half-chair* cyclohexene is more stable than the *boat* by 5.5 kcal mol^−1^ [140, 141], and for cyclooctane the boat–chair (BC) is more stable than twist-chair–chair (TCC) by 1.9 kcal mol^−1^ [142] (Fig. 6). Τhe DPLNO-CCSD(T) calculations calculate these conformational energies 5.97 kcal mol^−1^, 5.54 kcal mol^−1^ and 1.97 kcal mol^−1^. All theories calculate the *chair* cyclohexane as more stable than *twist-boat* while UFF and Dreiding force fields overestimating the energy by 2.85 and 1.70 kcal mol^−1^, respectively. In the case of cyclohexene all theories calculate the *half-chair* cyclohexene conformation as the global minimum but MMFF94 (2.06 kcal mol^−1^) and MM4-08 (1.73 kcal mol^−1^) deviate most from the DPLNO-CCSD(T) calculations. As regards cyclooctane, Dreiding and UFF force fields perform with the largest errors with the first force field calculating TCC and BC conformations with same energy and the second force field calculating TCC as more stable than the BC conformation by 2.41 kcal mol^−1^. From the other theories, the HF and B3LYP theories perform with the largest error (> 1.5 kcal mol^−1^) but the MMFF94 and Allinger’s force field perform better. For all the three molecules MP2 performed with the smallest deviation.

### Haloalkanes

**Fig. 7:**
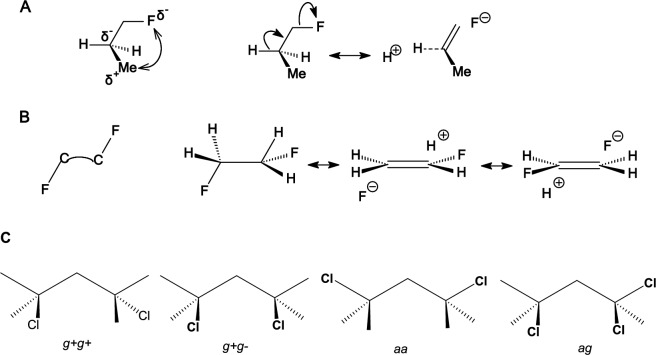
**A** Shows the stabilization of the *gauche* conformation by rotation about C2-C3 bond in 1-fluoropropane through attractive electrostatic interactions (left) and/or via hyperconjugative interaction (right). **B** Shows destabilisation of the anti conformer because of shaping bended bond C-C (left) and stabilisation of *gauche* conformation in 1,2-difluoroethane via hyperconjugative phenomenon (right). **C** Shows the conformarions of 1,3-dichloropropane

Τhe electronic effects of halogens, like electronegativity and hyperconjugation, impair force fields and other theories performance and this is more evident when two halogens are placed in proximal positions. Many of these factors were considered in MM4 force field parameters [143] to fix the deficiencies. Ιn halopropanes the experimental findings and previous MP2/6-311G(d,p) calculations [144] suggested that the conformer in which a methyl group is close to the halogen atom is favored due to attractive CH/*n* interactions [145]. The experimental measurements [146, 147] in the gas phase and previous calculations at the HF/6-31G+* level [148] and MP2/6-311G(d,p) level [144] showed that the *gauche* and *anti* conformations have equal energies in 1-chloropropane [144, 147] (Table 2) and the g*auche* conformer prevails as more decisive in 1-fluoropropane [144, 146, 148]. The stabilization of *gauche* with respect to the *anti* conformation may due to: (a) the hyperconjugative donation *σ*(C2-H) → σ*(C1-F) according to the resonance structures in Fig. 7) or/and (b) the favourable electrostatic interaction between induced dipoles (Fig. 7) [144, 148]. The errors in parameterization of MM3-96 for 1-fluoropropane and 1-chloropropane were corrected in the last versions of MM3 force field, e.g. in MM3-2000 [79, 149], and further in MM4 [143]; in these two latter force fields the electrostatic interactions are calculated considering dipole-dipole interactions beyond point charges. Our DLPNO-CCSD(T) calculations confirmed these preferences. Regarding the difference in energy between *gauche* and *anti* conformation for 1-fluoropropane the UFF, Dreiding, MM3-96 calculate clearly the wrong global minimum while MM+, MMX calculate almost equal energy (+ 0.08 kcal mol^−1^) for the two conformations as well as MMFF94 and HF/cc-pVTZ, HF/CBS (− 0.06, − 0.08, 0 kcal mol^−1^); MM3-00, MM4-08, HF/cc-pVDZ, B3LYP, MP2 calulate the correct *gauche* conformation as the global minimum. As regards the 1-chloropropane only MMFF94 and MP2 gave the right result providing equal energies for the *gauche* and *anti* conformation while MM3-96, MM3-00, HF/cc-pVDZ (+ 0.14 kcal mol^−1^) and MM4-08 (− 0.11 kcal mol^−1^) deviate less and UFF (+ 0.77 kcal mol^−1^), Dreiding (+ 0.52 kcal mol^−1^), HF/cc-pVTZ (+ 0.35 kcal mol^−1^) and HF/CBS (+ 0.41 kcal mol^−1^) deviate most.

The *gauche* conformation is preferred over *anti* in 1,2-difluoroethane as showed by experiments and DLPNO-CCSD calculations (Table 2) [150, 151]. This preference, which is also observed for other electronegative substituents, is known as the *gauche* effect. An explanation for this effect has been proposed on the basis of MP4/6-311++G(d,p) level calculations [152, 153] according to which: (a) the *anti* rotamer is destabilized [152] because in this position the *trans* electronegative substituents cause the C-C bond orbitals to bent in opposite directions resulting in bending geometry of the C-C bond (see left part of Fig. 7) and a weaker bond, whereas in the *gauche* rotamer the C-C bond orbitals bend in the same direction or/and (b) the *gauche* conformation is stabilized over competing electrostatic interactions between the fluorine atoms because of favouring hyperconjugative interactions *σ*(C-H) → σ*(C-F) [117] being possible due to the *gauche* position of fluorine substituents (see right part of Fig. 7). The opposite preference is observed experimentally in the gas phase in 1,2-dichloroethane [154] where *gauche* conformer is destabilized over *anti* due to the repulsion of bond C-Cl dipoles as showed by MP2/6-311++G** calculations [153]. While the cause of gauche conformation stability was also suggested as due to 1,3 C···F electrostatic polarization interactions that stabilize nearby carbon atoms [155] or similarly to electrostatic and exchange–correlation interactions [156] using state-of-the-art DFT calculations at theory level ZORA-BP86-D3(BJ)/QZ4P the rotational isomerism of 1,2-dihaloethanes XCH_2_CH_2_X (X = F, Cl, Br, I) was investigated as the interplay of hyperconjugation with Pauli repulsion between lone-pair-type orbitals on the halogen substituents that constitutes the causal mechanism for the *gauche* effect. Only in the case of the relatively small fluorine atoms, steric Pauli repulsion is too weak to overrule the *gauche* preference of the hyperconjugative orbital interactions. For the larger halogens, X⋅⋅⋅X steric Pauli repulsion becomes sufficiently destabilizing to shift the energetic preference from *gauche* to *anti*, despite the opposite preference of hyperconjugation [157, 158]. UFF, Deiding, MMX and HF/cc-pVDZ did not calculate the right preference for 1,2-difluoroethane while all other theories predict correctly the *gauche* conformation as more stable with MP2 showing the smallest deviation (+ 0.01 kcal mol^−1^) following by B3LYP (+ 0.12 kcal mol^−1^), MM4-08 (+ 0.12 kcal mol^−1^), MMFF94 (+ 0.15 kcal mol^−1^), MM+ (+ 0.14 kcal mol^−1^). For 1,2-dichloroethane all theories calculate the stabilization of *anti* over *gauche* conformation with UFF, Deiding or MM3-06 showing deviation ~ − 1 kcal mol^−1^ or + 1 kcal mol^−1^, respectively, following MM3-00 (− 0.44 kcal mol^−1^) or MMX (+ 0.49 kcal mol^−1^) while MP2 shows the smallest deviation (− 0.03 kcal mol^−1^). For 1,3-dichloropropane HF/6-31G(d) calculations [159] and B3LYP/6-31G(d) and MP2/aug-cc-pVDZ//B3LYP/6-31G(d) [160] suggest that as regard stability *g*^+^*g*^+^ *(*or *gg)* > *ag* > *aa* > *g*^+^*g*^*−*^ [112]. Ιn 1,3-dichloropropane [161] the *g*^+^*g*^+^ conformer is the global minimum stabilized with more favourable interactions between the two C-Cl dipoles compared to *g*^+^*g*^*−*^ conformer (see footnotes in Table 2 for definitions of these conformations). In a more recent study [162] using variable temperature infrared spectra of krypton solutions of 1,3-dichloropropane the enthalpy of the *ag* lies above the *g*+*g*+ by 0.78 kcal mol^−1^ which agree reasonable well with the the previously reported from the electron diffraction study [163] of 1.1 kcal mol^−1^ and 1.12 kcal mol^−1^ from wide-angle X-ray scattering [164]. In Ref. [162] the energy difference between the *aa* and *gg* conformations was measured as 1.09 kcal mol^−1^, while the previously obtained experimental [163] was ~ 1.5 kcal mol^−1^ and the calculated value using molecular mechanics [164] was 2.21 kcal mol^−1^, while it was shown also that B3LYP performed better than MP2 [162]. As shown in Table 2, UFF and DREIDING did not calculate *g*^+^*g*^+^, *ag*, *aa* with the correct ranking of minima whereas MM+ calculated all the minima having almost the same energy level. MP2 shows the smallest deviation (+ 0.12 kcal mol^−1^, + 0.05 kcal mol^−1^) followed by HF/cc-pVDZ (− 0.18 kcal mol^−1^, − 0.21 kcal mol^−1^), B3LYP (− 0.36 kcal mol^−1^, − 0.27 kcal mol^−1^) and MMFF94 (− 0.45 kcal mol^−1^, − 0.51 kcal mol^−1^). The remaining theories are HF/cc-pVTZ (− 0.65 kcal mol^−1^, − 0.43 kcal mol^−1^), HF/CBS (− 0.81 kcal mol^−1^, − 0.50 kcal mol^−1^) and the Allinger force fields MM3-96 (− 0.78 kcal mol^−1^, − 0.60 kcal mol^−1^), MM3-00 (− 1.17 kcal mol^−1^, − 0.76 kcal mol^−1^), MM4-08 (− 0.72 kcal mol^−1^, − 0.48 kcal mol^−1^) and MMX (− 0.88 kcal mol^−1^, − 0.71 kcal mol^−1^).

### Cyclohexane derivatives

**Fig. 8:**
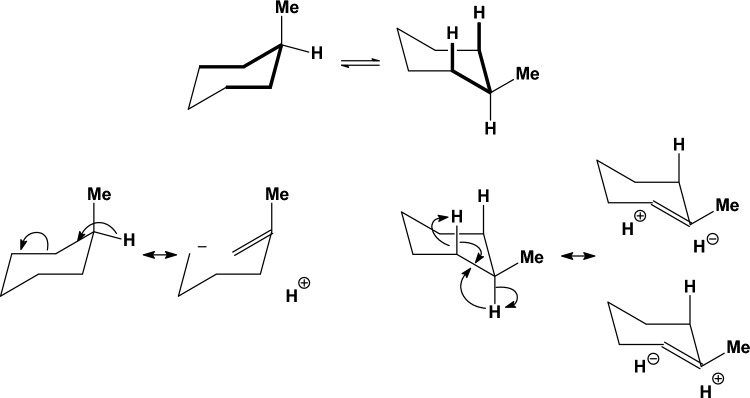
Top: equilibrium between low energy conformers in methylcyclohexane and the C-H bonds which participated (in bold) in the most important hyperconjugative interactions. Bottom: C-H bond participates in two hyperconjugative interactions in axial methylcyclohexane and in four hyperconjugative interactions in equatorial conformer

In monosubstituted cyclohexanes [112] (R = Me or i-Pr or t-Bu [165], Ph [166], Me_3_Si [167, 168], NH_2_ [169], OH or OMe [83], CO_2_Me or COMe [170], SH [171], PH_2_ [172], F [173, 174], Cl [175, 176], the equatorial orientation is lower in energy with citations included for the different substituents [177]. The stereoelectronic reasons for the higher stability of the equatorial over the axial (*ax*) conformations in monosubstituted cyclohexanes are still under investigation. The traditional model of 1,3-diaxial steric interactions between the axial substituent and the axial C3-H and C5-H bonds (steric *gauche* butane interaction between the axial substituent and carbons C3, C5) [165] provide a model adequate for most cases. However, compared to the synaxial repulsive interactions [165] model which destabilized the axial conformation compared to the equatorial (*eq*) conformation, it has been also proposed that the equatorial orientation is more stable than the axial orientation because of the stabilizing hyperconjugative σ_C-Hax_ → σ*_C-Hax_ interactions [178]. These include in the equatorial conformation the axial C-H bond of the carbon bearing the equatorial group and the axial C-H bond of the adjacent carbon [178] (Fig. 8). For groups with heteroatoms, X = N, O, F, Cl, electrostatic interactions stabilizing the *gauche* conformation in 1-fluoropropane or 1-propanol (Figs. 7, 12) are expected to stabilize also the axial conformation over equatorial [179]. Since the experimental data show that the equatorial conformer is the most stable in these cases [143], the previous effects dominate. In a selected group of substituted cyclohexanes the Δ*E*_ax-eq_, of monosubstituted cyclohexanes with OR (R = Me, Et, *i*-Pr and *t*-Bu) and R substituents (R = Me, Et, *i*-Pr and *t*-Bu) was calculated with HF, MP2 and QCISD theories with the 6-311G* and 6-311+G* basis sets [180]. The natural bond orbital method was applied to quantify the hyperconjugative contribution, Δ*E*_hyp_, to the relative stability of conformers. From the calculated values of Δ*E*_ax-eq_ and Δ*E*_hyp_ an estimate of the differential steric effect, Δ*E*_ster_, of substituents in cyclohexane was obtained. The values of Δ*E*_hyp_ and Δ*E*_ster_ show that they have a similar magnitude for OR substituents, while for R substituents the values of Δ*E*_ster_ are greater. The shift in the conformational equilibrium towards the axial conformer, the so-called anomeric effect, takes place when, within a series of substituents, hyperconjugative interactions and steric interactions balance in favour of the stability of this conformer. After our suggestion that axial substituents in cyclohexanes exert not only Pauli repulsion but also attractive LD interactions [129, 181, 182] and that DFT potential including the Grimme correction for LD interaction can be included for a more accurate description of Δ*E*_ax-eq_ systematic study using DLPNO-CCSD(T)/aug-cc-pVQ//B3LYP/def2-TZVP led to A-value scale that is can no longer be considered purely to arise from steric factors. Even for groups that do not participate in charge transfer or electrostatic interactions, the A-value includes Pauli repulsion and attractive LD interactions [183]. It has been observed with DNMR in solution an increase in population of axial conformer when passing from Me_3_SiO to the bulkier Ph_3_SiO group. An explanation was suggested for this effect, i.e. that is due to the increase in the attractive van der Waals interactions between SiR_3_ and axial CH bonds in the axial conformation; the number of these stabilizing interactions is larger in Ph_3_SiO-cyclohexane compared to the Me_3_SiO derivative [184]. Actually DLPNO-CCSD(T) calculations show that in the gas phase the axial conformation is more stable for Me_3_SiO while when this group is changed to Ph_3_SiO the axial and equatorial conformations become equal in energy which is the reversed from what is observed in solution [184].

MMFF94 fails in five compounds, Dreiding in three compounds, UFF, MM3-00, and strikinly also MP2 in two compounds, while MM+, MMX, MM3-93, MM4-08, HF/cc-pDVZ, HF/CBS, B3LYP only in one case. All force fields failed to calculate the axial conformer as the most stable one for Me_3_SiO group. Interestingly, all force fields, except Dreiding, as well as MP2 calculate fairly the increase in population of axial conformer when passing from Me_3_SiO to the bulkier Ph_3_SiO group. UFF have the largest deviations being > 1.5 kcal mol^−1^ in 3 cases and > 1 kcal mol^−1^ in 1 case. Interestingly all HF theories have a deviation > 1 kcal mol^−1^ for phenylcyclohexane. Compared to DLPNO-CCSD(T) reference energy values the experimental results disagree for the Me_3_SiO group.

**Fig. 9:**
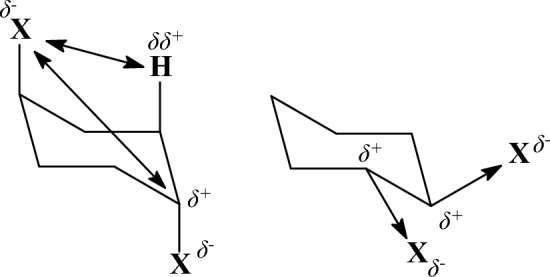
Diaxial conformations of 1,4-dihalo cyclohexane (left) and di-equatorial conformation in 1,2-dihalo cyclohexane (right)

In *trans*-1,2-dihalogen cyclohexanes, the di-equatorial is destabilized because of the repulsive interactions between the C-X dipoles compared to the di-axial conformation, while the di-axial conformation is destabilized because of the Pauli repulsion between axial C-X and axial C-H bonds which is particularly important in the *trans*-1,2-dichloro and *trans*-1,2-dibromo derivatives compared to the *trans*-1,2-difluoro because of the bigger size of bromine and chlorine over the not significant size of fluorine [185]. However, in the diaxial conformation also attractive interactions exist between axial C-X dipoles and between axial C-X dipoles and axial C-H bond (Fig. 9).

The electrostatic repulsion between the C-F dipoles is larger in the di-equatorial *trans*-1,2-difluoro cyclohexane and the equilibrium is more shifted to the di-axial conformation which has a 54% population as was shown by experimental measurements in the gas phase with electron diffraction [186] and QCISD/6-311+G(2df,p) calculations while in solution the diequatorial predominates for the *trans*-1,2-dihalogen cyclohexanes [187, 188]. Thus, the conformational preference is not the same as in *trans*-1,2-difluoroethane where the gauche conformer is preferred over the *anti* as previously discussed in haloalkanes [150, 151].

In the *trans*-1,2-dichloro the experimental measurements in the gas phase [186] and the CCSD/6-311+G(2df,p) calculations [186] show that the diaxial has an 60% population as the Pauli repulsion between axial C-X and axial X-H bonds cannot destabilize the diaxial over the diequatorial conformation.

The experimental data [189] from electron diffraction in the gas phase for 1,4-dichlorocyclohexane and high precision QCISD/6-311+G(2df,p)//MP2/6-311G(d) calculations in 1,4-dichlorocyclohexane (which are performed to reproduce reliably the gas phase behaviour) suggested that the two conformers have equal stability. The calculations with theories HF/6-31G*, MP2/6-311G*, QCISD/6-311+G(2df,p), MPW1PW91/6-311G*, B3P86/6-311G*, B3P86/6-311+G(2df,p) were also tested showing that the results for Δ*Ε*_ax-eq_ with HF/6-31G* are poorest. The experimental composition is most accurately predicted by the MP2/6-311G* and QCISD/6-311+G(2df,p) calculations from the conformational energy differences. According to the QCISD/6-311+G(2df,p)//MP2/6-311G(d) calculations in the trans-1,4-dihalocyclohexanes [187], the small conformational preference for the dichloro and dibromo compounds probably results from a competition between the normal equatorial preference (+ 0.1 kcal mol^−1^), and the Coulombic attraction between C-X dipoles in the diaxial form. Fluorine has a smaller equatorial preference than Cl or Br, and the larger C-F bond dipole will lead to a larger attraction in the diaxial form with an − 1.1 kcal mol^−1^ [187]. The combination of these two factors results in a strong calculated diaxial preference for trans-1,4-difluorocyclohexane.

Our DLPNO-CCSD(T) reveal than in all four the diaxial is lower in energy compared to diequatorial. For the *trans*-1,2-difluoro-cyclohexane or *trans*-1,2-dichloro-cyclohexane the corresponding percentages from our calculations are 50.1 or 54% compared to 54% (− 0.1 kcal mol^−1^) or 60% (− 0.3 kcal mol^−1^), respectively, from experiments and QCISD calculations [187].

The diequatorial substitution is also observed in 1,2-dimethylcyclohexane [190], albeit less pronounced because of the steric repulsion between the *gauche* methyl groups. In 1,3-dimethylcyclohexane [191] the preference for the diequatorial conformer equilibrium returns to the common value since the two methyl groups are now apart enough to interact seriously. In the trans-1,2-bis(trimethylsilyl)cyclohexane the diaxial conformer is more stable than the diequatorial conformer because of the severe steric repulsion in the last and also due to the LD attractive interaction of axial SiMe_3_ groups [192]. For the *trans*-1,2-dimethylcyclohexane and *cis*-1,3-dimethylcyclohexane all theories calculated the right global minimum, i.e. the diequatorial over diaxial conformation with UFF and HF theories showing deviation > 1.5 kcal mol^−1^. Dreiding and MMFF94 performed with deviation > 3 kcal mol^−1^ and HF theories with a deviation > 1.5 kcal mol^−1^. As regards the four *trans*-dihalogen cyclohexanes, Dreiding and UFF calculate the wrong global minimum in all four molecules examined with strong deviation for three out of the four cases, MM+, MM4-08, HF/CBS failed to predict the right global minimum in two cases while MM3-00, HF/cc-pVTZ, and B3LYP in just one case.

### Oxygen-containing compounds

We performed calculations in important categories of oxygen-containing compounds, i.e., carboxylic acids, esters, aldehydes, ketones, alcohols, ethers, acetals (Table 4). In formic acid [193–195], carboxyl group adopts two distinct planar geometries in rare gas matrices at low temperature and prefers a *Z*- or *syn*-conformation in which the C=O and O-H or O-CH_3_ bonds are in eclipsed orientation. In the formic acid the O-H group is oriented at ∼ 60° with respect to the C═O in the gas phase and in the* E*- or *anti*-conformation the O-H is antiparallel to the C═O. The *Z*(*syn*) is more stable by 3.90 kcal mol^−1^ in formic acid [196] according to microwave spectroscopy. The *Z* conformation of methyl formate has been found to be 4.8 kcal mol^−1^ more stable than the *E* form, and with methyl acetate the energy difference was found to increase to 8.5 ± 1 kcal mol^−1^ [197]. Methyl formate has been also studied with IR and by DNMR and the free energy difference with the latter method has been determined to be 2.15 kcal mol^−1^ in an apolar solvent [198, 199]. Using femtosecond 2D-IR spectroscopy [200] it was demonstrated that formic acid adopts the two distinct, long-living conformations *syn* and *anti* in deuterated acetonitrile and heavy water solutions, The fractions of the *anti*-conformation and the *syn*-conformation are 20–30% and 80–70%, respectively, both in deuterated acetonitrile and in heavy water solutions. The distinct conformers of the carboxylic acid and their slow exchange at room temperature shows that these conformers are separated by high energy barriers. As a result, the presence of these conformers can have a large effect on the structure and dynamics of (bio)molecular systems. Similar conformational behaviour exist for methyl formate [201] or methyl acetate studied also in the gas phase [197, 202]. In solution formate species have been studied by DNMR [203]. The considerably higher energy content of 8.5 kcal mol^−1^ [197] in *E*(*anti*) conformation in methyl acetate is due to proximity of methyl groups. In ethyl acetate in the *E*(*anti*) conformation around (O=)Csp^2^‒OCH_2_CH_3_ rotor the *eclipsed* and *skew* con formations depending if the methyl or C-H groups of ethyl groups are eclipsed as regards the C=O bond.

The experimental results for propanal [204] or 2-butanone [205] show that the global minimum corresponds to an *eclipsed* orientation of carbonyl bond and 3- or 4-methyl groups, respectively, to avoid steric repulsion between methyl groups in the *skew* conformation with relative conformational energies 0.95 or 2.0 kcal mol^−1^. MP2/6-311G(d,p) calculations [206] suggest that for 2-butanone or propanal, the Pauli repulsive and the bond dipole interactions are primarily responsible for the conformational preference of the *skew* (*gauche* in Ref. [205]) by 1.81 kcal mol^−1^ or 1.22 kcal mol^−1^, respectively, over the *eclipsed* in good agreement with experimental [205] and other computational results [207, 208].

**Fig. 10:**
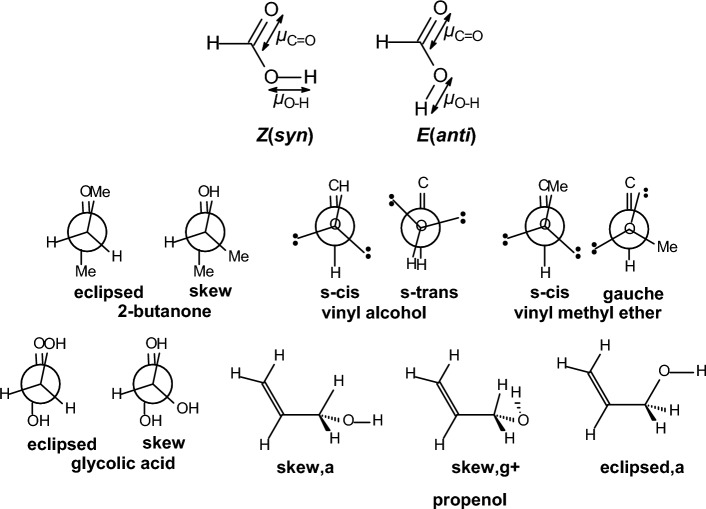
Low energy conformers of 2-butanone, vinyl alcohol, vinyl methyl ether, glycolic acid and propenol

Similarly, based on electron diffraction data [209–211, 212] and calculations [211, 213–215] glycolic acid prefers a global minimum in which C=O bond is *eclipsed* to O-H bond corresponding to the *skew* conformation in which a hydrogen bond is formed between the hydroxyl proton and carbonyl rather than having C=O bond *eclipsed* to the C-O bond in the *eclipsed* conformation with a hydrogen bond between the alcohol hydrogen and a lone pair of the carboxylic acid hydroxyl group. The electron diffraction data overestimate the energy difference to 4.2 kcal mol^−1^ [209–211] compared to the 2.51 kcal mol^−1^ from MP2/6-311++G(2d,2p)//MP2/6-31G(d,p) or 2.71 kcal mol^−1^ from MP2/cc-pVQZ//MP2/cc-pVTZ and CCSD(T)/6-31G(d,p)//MP2/cc-pVTZ calculations (Fig. 10) [211, 213]. Α similar conformation global minimun is adopted by glycolic methyl ester. ^69,197–201^ Propenol adopts the *skew*,*g*+ conformation as the global mimimum according to the gas phase electron diffraction [216], microwave [217] and infrared data in combination and HF calculations [216] (Fig. 10).

The vinyl alcohol [218] and methyl vinyl ether [219–224] were investigated experimentally and by calculations [225–227]. For methyl vinyl ether the *s*‐*cis* conformer was more stable than the *gauche* conformer (torsional angle 114°) by approximately 1.15 kcal mol^−1^ [219] as measured experimentally in the gas phase. The MP2/6-31G*//MP2/3-21G calculations of vinyl alchohol produced a relative energy of 2.08 kcal mol^−1^; the experimental estimate [219] agrees better with B3LYP/6-311++G(2d,2p)-optimized value (1.19 kcal mol^−1^) [227].

UFF, Dreiding force fields failed to predict the right global minimum in all cases and B3LYP6-31G(d,p) in one case. MM+ force field deviate in two cases by more than 3 kcal mol^−1^ while MMX force field deviate in one case by more than 3 kcal mol^−1^ and one case more than 1.5 kcal mol^−1^ while HF/cc-pVDZ theory deviate in one case by more than 1.5 kcal mol^−1^ and MP2 theory perform best.

**Fig. 11:**
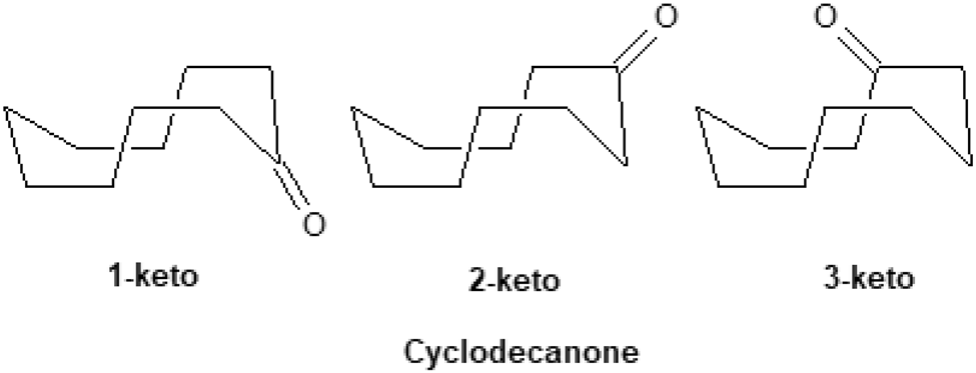
Conformations of cyclodecanone

In cyclohexanone the chair conformation is the global minimum and in the 2-methylcyclohexanone methyl group prefers the equatorial over the axial position by 1.58 kcal mol^−1^ according to dynamic NMR in solution [228]. In cyclodecanone the carbonyl group defines three different conformations, the *1-keto*, *2-keto* and *3-keto* conformations (Fig. 11). According to the dynamic NMR data and X-ray crystallography studies [229, 230], cyclodecanone adopts the *3-keto* conformation (Fig. 11). The 1-keto and *2-keto* conformations are higher in energy because of the higher number of neighbouring C-H···H-C repulsive interactions as has been suggested using MM3-96 force field calculations [231]. We calculated the conformational energies *ax-eq* and *1keto-3keto*, *2keto-3keto* for the 2-methylcyclohexanone and cyclodecanone, respectively. UFF and Dreiding force fields calculate the equatorial methyl conformation of 2-methylcyclohexanone with higher energy than axial conformation and UFF deviate in *1keto-3keto* energy difference with a deviation > 1.5 kcal mol^−1^. MP2 theory performed with the smaller deviations (+ 0.10 or + 0.26 kcal mol^−1^ and + 0.65 kcal mol^−1^, respectively) compared to the DLPNO-CCSD(T) values following B3LYP (+ 0.18 or − 0.12 kcal mol^−1^ and − 0.58 kcal mol^−1^, respectively) and then MM4-08 (+ 0.35 or − 0.08 kcal mol^−1^ and − 0.30 kcal mol^−1^, respectively) and MMFF94 (− 0.42 or − 0.10 kcal mol^−1^ and − 0.02 kcal mol^−1^, respectively).

Ethanol [232, 233] and ethyl methyl ether [234] have been studied in the gas phase as mixture of *anti* and *gauche* conformations. For ethanol, calculations have been performed at the MP2/aug-cc-pVTZ, CCSD(T)/aug-cc-pVTZ or aug-cc-pVQZ theories  [235] along with various other theories; the *anti* conformation was calculated theory to be 0.13 kcal mol^−1^ more stable compared to the *gauche* conformation using CCSD(T)/aug-cc-pVQZ theory [235] in excellent agreement with the experimental value of 0.129 kcal mol^−1^ [232] which is close to our 0.16 kcal mol^−1^ using DLPNO-CCSD(T) calculations. For ethyl methyl ether also various levels of theory have been applied, e.g. MP2 [236, 237] or CCSD, QCISD or CCSDT, QCISDT [237] with various basis sets which prodided energy values 1.38 or 1.36, 1.34 kcal mol^−1^ or 1.30, 1.30 kcal mol^−1^ using 6-31G(d) basis set [237] which are also very close to our calculated 1.30 kcal mol^−1^ with DLPNO-CCSD(T) theory.

2-Propanol has three minima, i.e., (+/−)-*gauche* and *anti*, that are defined by its hydroxyl orientation [238, 239]. Previous MP2/aVTZ//MP2/VDZ [239] and more recent CCSD(T)/aVTZ//MP2/aV5Z [240] calculations predicted that the (+/−)-gauche to be 0.257 kcal·mol^−1^  [240] more stable than the *anti* conformation, that lie in the middle of the experimental range of 0.025–0.450 kcal·mol^−1^ [238, 241].

The experimental information regarding the relative stability of 1-propanol’s minima, by rotation of C2-C3 bond, is unclear because of vague identifications and contradictions within the literature [242–244]. However, MP2/aVTZ//MP2/VDZ [239] calculations clearly predicted that the pair of the two *gauche* enantiomeric conformations correspond to the global minima [239]. According to these calculations [239] and recent CCSD(T) calculations [240] the *anti* conformation has slightly increased energy by 0.11–0.13 kcal mol^−1^ and our DLPNO-CCSD(T) calculations provide a value of 0.10 kcal mol^−1^.

**Fig. 12:**
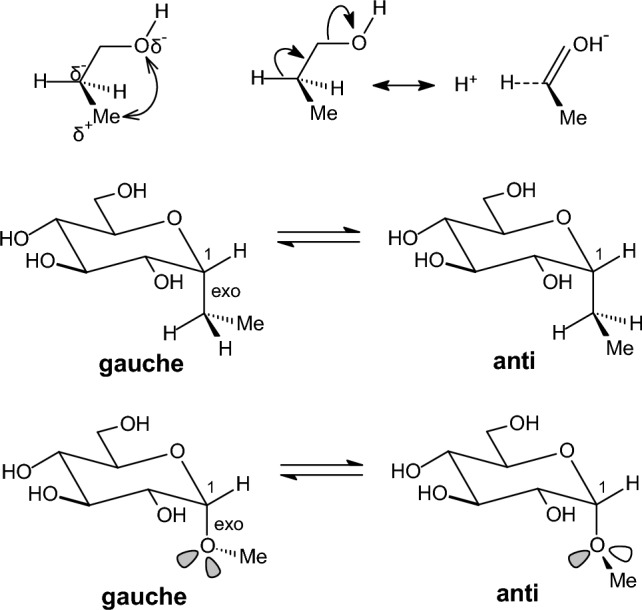
Top: stabilization of *gauche* conformation of 1-propanol over *anti* conformation by rotation of C2-C3 bond is likely due to electrostatical interactions (left) and hyperconjugative interactions (right). Middle and bottom: the same conformational preferences are observed for the O-C1-C_exo_-C dihedral in C- and O-glycosides

According to HF/6-31+G(d) calculations the stabilization (by 0.3 kcal mol^−1^) [143, 245] of the *gauche* conformation over *anti* can be attributed to the attractive electrostatical interaction, shown in the first line in the left-hand part of Fig. 12, since the C^*δ*+^-Ο^*δ−*^ dipole induces an excess positive charge at the hydrogen atoms of methyl C-H bonds resulting in attractive interactions. This attractive interaction counterbalances the steric repulsion between OH and CH_3_ groups in the *gauche* conformer. Additionally, in the *gauche* conformation the hyperconjugative interaction *σ*(C2-H) → σ*(C_1_-O) with a second-order perturbation energy 4.42 kcal mol^−1^ contribute to the stabilization of *gauche* conformation (Fig. 10, first line, right hand part).

All theories predict correctly the correct global minimum for ethanol, methyl ethyl ether, 2-propanol, i.e. the *anti* conformation, is more stable than the *gauche* conformation. The MP2 and B3LYP theories have the smallest deviation from DLPNO-CCSD(T) following by HF/cc-pVTZ, HF/CBS, MM4-08, MMFF94. The biggest deviation was observed for UFF and then MM3-96, MM3-00 and the MM2 analogs MM+ and MMX.

As regards 1-propanol, all the force fields fail to predict the correct global minimum, i.e. the *gauche* conformation. Ηowever, using the MM4 force field the correct conformational preference in 1-propanol is calculated since MM4 includes terms to account for the induced dipoles [80] compared to MM3 [246]. More accurate parameters relative to the C-C-O angle bending and the barrier of the C-O bond rotation were included in MM4 compared to MM3 [246]. Additionally, HF/cc-pVDZ and MP2 calculate correcty the gauche conformation as global minimum while B3LYP and HF/cc-pVDZ predict *gauche* and *anti* conformations with equal energy.

According to experimental ^3^*J* (^1^H-^1^H) values the same conformational preference of *gauche* relative to *anti* conformation, as regards the O-C1-C_exo_-C dihedral angle, is observed in the C- and O-glycosides (see second and third lines in Fig. 12). The calculations in Table 4 for the O-Et glycoside, as regards the comdormations that generate as regards torsion O-C_1_-C_exo_-C, show that all theories tested calculate the correct conformation, with UFF and MMFF94 deviating the most, followed by HF (Table 4).

2-Propen-1-ol (allyl alcohol) has been investigated in the gas phase [216, 247], with MP2/cc-pVTZ or B3LYP calculations [247] and MP4/TZP//MP2/6-31G* [38]. Conformation *sk,g*+ is the global minimum following by *ecl,a* and *sk,a* conformations (Fig. 10). All force fields, except MMFF94 which was parameterized using very accurate ab initio calculations [38], failed to calculate correcty the energy ranking according to the reference DLPNO-CCSD(T) calculations and HF calculations deviate the least, following the MP2, MMFF94 and then B3LYP. The MP4/TZP//MP2/6-31G* reported in the literature are close to the DLPNO-CCSD(T) values.

**Fig. 13:**
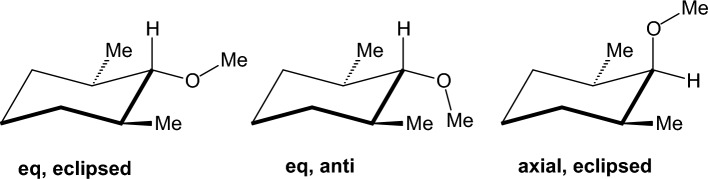
The buttressing effect of methyl groups forces the O-Me group in *cis*-2,6-dimethyl-1-methoxycyclohexane eclipsing tertiary C-H bond

The buttressing effect of the two equatorial methyl groups in the cis-2,6-dimethyl-1-methoxycyclohexane favors an *eclipsed* conformation by rotation around C-O bond (Fig. 13) placing hydrogen and methyl groups in *eclipsed* position [248]. The MM+, MMX, MM3-96, MMFF94 calculate the *anti* compared to the *eclipsed* conformation as the global minimum. Big deviations are calculated with UFF (− 5.06 kcal mol^−1^), HF theories have > 1 kcal mol^−1^ deviation and the smallest deviation was observed in MP2 (+ 0.01 kcal mol^−1^) following by Dreding (+ 0.17 kcal mol^−1^), B3LYP (− 0.54 kcal mol^−1^) and then MM3-00 (− 0.62 kcal mol^−1^), MM4-08 (− 0.58 kcal mol^−1^).

**Fig. 14:**
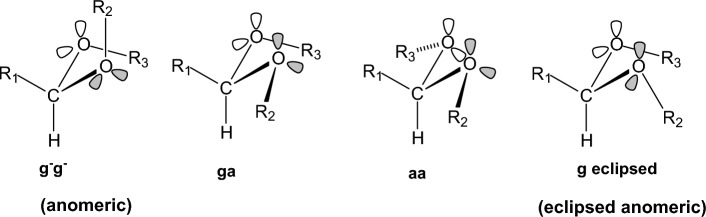
Conformations around central C-O bonds in acetals R_1_CH(OR_2_)(OR_3_)

Dimethoxymethane, which is the dimethyl acetal of formaldehyde, prefers the *gauche* conformation around central C-O bonds, i.e. the anomeric *g*^*−*^*g*^*−*^ conformation compared to the *anti* conformation (Fig. 14, R1 = H, R_2_ = R_3_ = Me). The steric repulsive *gauche* interaction is compensated by the two anomeric interactions. The anomeric interaction is defined as the increased stabilization resulting if a non-bonding electrons pair of heteroatom has an antiparallel orientation with respect to a polarized C-O bond. In dimethoxymethane, there are two such anomeric interactions, each including a non-bonding electrons pair in one oxygen with an antiparallel orientation with a polarized C-Ο bond. It has been explained that this preference is observed as the result of minimization of the repulsive interactions between C-Ο dipoles and electron pairs.

The anomeric *g*^*−*^*g*^*−*^ conformation is known to be the global energy minimum form of dimethoxymethane, according to a number of experiments employing electron diffraction [249, 250], nuclear magnetic resonance [251], X-ray diffraction [252], infrared spectroscopy in argon matrices [253], or rotational spectroscopy [254, 255] and ab initio or DFT calculations [253, 256, 257] with the more recent at CCSD(T)/aug-cc-pVDZ//B3LYP/aug-cc-pVTZ level [257]. This conformational preference is due to the hyperconjugative interaction *n*(O) → σ*(C-Ο). In terms of resonance structures this lone pair electrons donation can be described with the structures C-Ο-C-Ο-C ↔ C-Ο^+^=C^−^Ο-C [244, 258]. Additional experiments and ab-initio calculations suggested that the preference for the anomeric *g*^*−*^*g*^*−*^ conformation is due to attractive C-H⋯O interactions [129, 182, 259–261], e.g. in *g*^*−*^*g*^*−*^ conformation there are two *gauche* attractive interactions between oxygen lone pairs and C-H bond but in *ga and* aa conformations there is only one [259]. The anomeric conformation is the global minimum also for acetaldehyde dimethylacetal according to Cambridge Crystallographic Database and of molecular mechanics calculations, and by NMR measurements of simple model acetals [262, 263]. Results based on coupling constants ^1^*J*_C-H_, ^*3*^*J*_C-H_ showed that for acetals R_1_CH(OMe)_2_ the common anomeric conformation is quickly destabilised as R_1_ increases in size [263]. The steric *gauche* interaction between groups R_1_ and OR_2_ forces group OR_2_ to eclipse C-H bond (Fig. 12) through rotation by ~ 180° since in the new *g,eclipsed* conformation the two anomeric (hyperconjugative) interactions are maintained. Thus, while the formaldehyde dimethylacetal, i.e. the dimethoxyethane (R_1_ = H, R_2_ = R_3_ = Me), adopts the standard anomeric conformation *g*^*−*^*g*^*−*^, the *g,eclipsed* conformation is considerably populated in acetaldehyde dimethylacetal (R_1_ = R_2_ = R_3_ = Me) and is the global minimum for bigger alkyl groups, e.g. when R_1_ = *i*-Pr, *t*-Bu.

For formaldehyde and acetaldehyde dimethyl acetal UFF and Dreiding force fields failed to calculate correctly the anomeric effect and both calculate as global minimum the *aa* conformation for the former compounds and UFF the *g eclipsed* for the latter. MM+, MMX force fields perform with significant deviations. Most accurate are B3LYP and MP2 theories with next the HF/cc-pVDZ theory then the MM4-08 force field following by the other theories.

Because of the anometic effect [264], the axial conformation of 2-methoxytetrahydropyran and 2-fluorotetrahydropyran is favoured by 1.27 and 2.45 kcal mol^−1^, respectively, over the equatorial according to the accurate CCSD(T)/aug-cc-pVDZ//MP2/6-311G(2df,2pd) calculations [265]. The conformational preferences of 2-methoxytetrahydropyran have been investigated using Dynamic NMR [266] and calculations [267–269] and for 2-fluorotetrahydropyran experimental and calculated ^1^*J*_C-F_ values and conformational energy calculations have been used [270–272] and calculations. Our DLPNO-CCSD(T) calculations show this difference to be 1.21 and 2.43 kcal mol^−1^, respectively. All the force fields, except UFF and Dreiding, predict the stabilization of axial conformer for MeO-THP and F-THP but Allinger force fields, their clones (MM+, MMX) and MMFF94 performed with deviations > 1.5 kcal mol^−1^ in the case of 2-fluorotetrahydropyran. In the case of 2-methoxytetrahydropyran the MM4-08 performed accurately (− 1.26 kcal mol^−1^). For both 2-methoxytetrahydropyran and 2-fluorotetrahydropyran, MP2 show the smallest deviations (− 1.40 kcal mol^−1^, − 2.44 kcal mol^−1^) following HF/cc-pVDZ (− 1.28 kcal mol^−1^, − 2.73 kcal mol^−1^) and B3LYP (− 0.78 kcal mol^−1^, − 2.93 kcal mol^−1^). The anomeric effect favours the axial conformation in O-glycosides [258]. Additionally, it is noted that the *gauche* conformation is adopted as regards the dihedral angle O-C-O_exo_-C in O-glycosides (Fig. 12). This preference is characterized as exo-anomeric effect and is also observed in C-glycosides [273]. All theories calculate the correct minimum but most of them overestimate conformational energy.

The conformational energy of 2-methyltetrahydropyran is higher than that of methylcyclohexane because the smaller length of C-O bond forces the axial methyl to be in closer distance with the axial C-6 hydrogen. In 4-methyltetrahydropyran the conformational energy value is similar to that of methylcyclohexane since the smaller in length C-O bond does not affect the distance between axial Me and axial H in 1,3-positions. In 3-methyltetrahydropyran the destabilization of the axial conformer is smaller compared to the 4-methyl analogue, since the synaxial 1,3-Me⋯H is replaced by the synaxial Me⋯Lp [178] for which Pauli repulsion is less. All theories calculate these preferences. Between all theories tested MP2 shows values closer to the DLPNO-CCSD(T) calculations, followed by MM3-96, MM3-00, MM+, MMX.

**Fig. 15:**
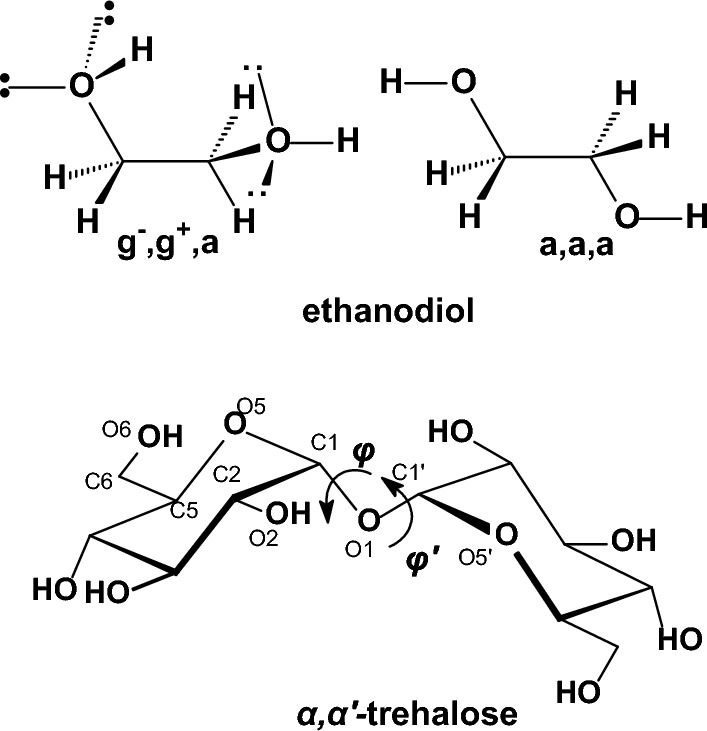
Conformations of 1,2-ethanediol and description of the conformarional space of trehalose by rotation around dihedral angles *φ* and *φ*′

Among the tested compounds having two functional groups (Table 4) ethanediol has two vicinal hydroxyl groups (Fig. 15). A large number of ab initio studies have been carried out in the gas-phase for ethanediol, ranging from HF calculations and partially optimized geometries to G2(MP2) calculations with fully optimized MP2/6-31+G* geometries [274–276]. All of these investigations found that the relative energies of all 10 rotamers lie within 3.49 kcal mol^−1^, with the *g*^*−*^*g*^+^*a* isomer being the lowest in energy (Fig. 15). These theoretical results are in good agreement with experimental results [277, 278]. According to οur reference DLPNO-CCSD(T) calculations the *g*^*−*^*g*^+^*a* conformation is the global minimum stabilized by the formation of a hydrogen bond between the hydroxyl groups. UFF and Dreiding are the only theories that calculate the *aaa* isomer instead of the *g*^*−*^*g*^+^*a* conformation while MMX calculate *g*^*−*^*g*^+^*a* and *aaa* conformations with equal energy. MM + is the next worst with deviation − 1.81 kcal mol^−1^ then HF/cc-pVTZ and HF/CBS with deviation − 0.88 kcal mol^−1^ and − 1 kcal mol^−1^, MM3-00 and HF/cc-pVTZ with deviations − 0.53 and − 0.49 kcal mol^−1^, respectively. MM4-08, B3LYP and MP2 have the best performance with deviation − 0.3, + 0.07 and + 0.27 kcal mol^−1^, respectively.

We also performed calculations on the disaccharide *a,a*-trehalose (α-d-glycopyranozyl-1,1-α-d-glycopyranoside) to study conformations of trehalose by rotation around dihedral angles *φ* and *φ*′ (Fig. 15). The connection of the two monosaccharides in the disaccharide in Fig. 15 is 1 → 1 glycosidic bond, with two axial anomeric bonds. The symmetry of the disaccharide around glycosidic oxygen drastically limits the conformational space. The conformational preferences of this molecule are valuable for the conformational analysis of polysaccharides. According to previous B3LYP/6-31G(d) calculations, which are reliable for reproducing gas phase conformational energies of disaccharides [107], three conformations are conformational minima arising from different combination of *φ* and *φ*′ dihedral angles, i.e. The gtxgtx16080 (*φ* = 160, *φ*′ = 80), the gtxgtx6060 (*φ* = 60, *φ*′ = 60) and the tgctgr180180 (*φ* = 180, *φ*′ = 180) conformations with gtxgtx16080 being the global minimum [107]. The dihedral angles values for gtxgtx16080, gtxgtx6060 and tgctgr180180 conformations after our energy minimization with B3LYP/6-31G(d,p) are (*φ* = 157, *φ'* = 83), (*φ* = 65, *φ'* = 65), and (*φ* = 177, *φ'* = 176), respectively, in agreement with the literature data [107]. Previous calculations with the MM3 force field [279], also show that gtxgtx6060 (*φ* = 60, *φ'* = 60) conformation is the global minimum. Our reference CCSD(T)-DPLNO calculations confirmed that gtxgtx16080 conformation (*φ* = 160, *φ*′ = 80) is almost equal in energy with gtxgtx6060 which is observed in the solid state; gtxgtx16080 is the global minimum with slightly higher energy by 0.04 kcal mol^−1^ compared to gtxgtx6060. Only MP2 and B3LYP calculate correctly the conformational preferences, i.e. tgctgr180180 has much higher energy (6.96 kcal mol^−1^) than gtxgtx6060 and gtxgtx16080. Thus, MM+, MMX, MM3-96, MMFF95 calculate tgctgr180180 only 0.4–1 kcal mol^−1^ higher than gtxgtx16080 while UFF, Dreiding suggest that tgctgr180180 has lower energy compared to gtxgtx16080. Then all theories calculate that gtxgtx6060 is clearly the global minimum having by ~ 1.2 (MMFF94) − 7.4 kcal mol^−1^ (MM+) lower energy than gtxgtx16080 and only HF theories by only 0.3–0.76 kcal mol^−1^. MM3 and MM+ force fields did not calculate gtxgtx16080 and tgctgr180180 as stable conformations.

### Nitrogen-containing compounds

The preferred conformation of the lp-N-C-C moiety for the aliphatic amines is varied. Several experimental studies has been performed for ethylamine in the gas phase combined with ab initio calculations [280, 281] while calculations at G3MP2B3 and G3B3 levels of theory suggested that the *anti* and *gauche* conformations differ only by ~ 0.063 kcal mol^−1^ [282]. Indeed our DLPNO-CCSD(T) calculations show almost equal in energy conformations with a difference in energy of 0.09 kcal mol^−1^. The experiments in the gas phase showed that for 1-propylamine [283, 284] both the *Tt* and *Gt* conformations have been detected and for 2-propylamine the *anti* and *gauche* conformations have been detected [284, 285]. The CCSD(T)/aug-cc-pVTZ//MP2/aug-cc-pVTZ calculations revealed that in propylamine the *Tt* conformation is the most stable one, followed by *Tg* (0.048 kcal mol^−1^) conformation while in 2-propylamine the *anti* conformation is preferred over *gauche* conformation by 0.430 kcal mol^−1^ [284]. Also experimental data in the gas phase detected as global minimum for methylethylamine the *Tg* conformation following by *Gt* and *Gg* supported by B3LYP/6-311+G(d,p) [286]. In piperidine [287–289] or pyrrolidine [290, 291] a double *gauche* conformation is preferred, corresponding to an axial lp and equatorial N-H group in pyrrolidine. The CCSD(T)/aug-cc-pVTZ//MP2/aug-cc-pVTZ calculations [290] suggest a 0.0486 kcal mol^−1^ preference for the equatorial N-H group in pyrrolidine. Obviously, Dreiding, UFF, MMFF94 deviate significantly (~ − 0.4 to − 0.6 kcal mol^−1^) from the equal in energy conformations result for ethylamine following the MM4-08 (− 0.12 kcal mol^−1^). The deviations of MM+ (− 0.04 kcal mol^−1^), MMX (+ 0.07 kcal mol^−1^), MM3-96 (− 0.01 kcal mol^−1^), MM3-00 (− 0.04 kcal mol^−1^), HF (− 0.07 to + 0.17 kcal mol^−1^), B3LYP (+ 0.16 kcal mol^−1^) and MP2 (− 0.09 kcal mol^−1^) are small and all the theories provide values close to the DLPNO-CCSD(T) value. For 1-propylamine the *Gt* and *Tt* conformations, for pyrrolidine the *E*(2)N-H *ax* and *eq* conformations and for hexahydropyrimidine the NH,NH *ax,eq* and *ax,ax* conformations are similar in energy. In all three cases MP2 with a deviation + 0.16 kcal mol^−1^, + 0.08 kcal mol^−1^ and + 0.08 kcal mol^−1^, respectively, performed accurately while for pyrrolidine the MMX has a very small deviation (+ 0.02 kcal mol^−1^) and for hexahydropyrimidine the B3LYP (+ 0.08 kcal mol^−1^). For piperidine the MP2 (+ 0.07 kcal mol^−1^), B3LYP (− 0.02 kcal mol^−1^), HF/cc-pVDZ (+ 0.13 kcal mol^−1^), MMFF94 (+ 0.12 kcal mol^−1^) and UFF (− 0.01 kcal mol^−1^) performed accurately. For methylethylamine strikingly the MP2 (+ 0.59 kcal mol^−1^) does not perform well while MMX (+ 0.16 kcal mol^−1^) and MM4-08 (− 0.12 kcal mol^−1^) performed better. For 2-propylamine MP2 (− 0.07 kcal mol^−1^), B3LYP (− 0.12 kcal mol^−1^), HF/cc-pVTZ (− 0.07 kcal mol^−1^), HF/CBS (0 kcal mol^−1^), MM4-08 (− 0.14 kcal mol^−1^), MMFF94 (− 0.01 kcal mol^−1^) performed accurately.

**Fig. 16:**
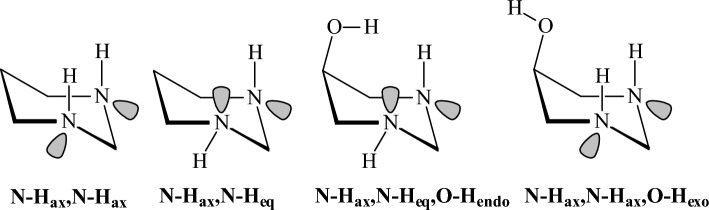
Conformations of hexahydropyrimidine and of its 3-OH analogue

The conformations of hexahydropyrimidine and of the 3-OH analogue differ in the orientation of the N-H group (Fig. 16); in 3-OH hexahydropyrimidine a hydrogen bond can stabilize N-H_ax_,N-H_eq_,O-H_endo_ over the H_ax_,N-H_ax_,O-H_exo_ conformation [104]. Since, the energy changes between the two conformations from electrostatic and electronic effects are marginal, 3-OH hexahydropyrimidine is a good model for testing the performance of different theories in the calculation of the conformational energy. Compared to our reference DLPNO-CCSD(T) calculations, as regards the 3-OH hexahydropyrimidine, from the force fields used, only MM3-96 (+ 2.33 kcal mol^−1^), MM3-00 (+ 0.95 kcal mol^−1^), MM4-08 (+ 3.01 kcal mol^−1^) and MMFF94 (+ 2.83 kcal mol^−1^) calculate the N-H_ax_,N-H_eq_,O-H_endo_ as more stable conformation compared to the H_ax_,N-H_ax_,O-H_exo_ conformation but with MM3-96 or MM3-00 and MMFF94 performing with big deviations, > 1.5 kcal mol^−1^ or > 3 kcal mol^−1^, respectively as noted inside the perenthesis. MP2 (+ 0.2 kcal mol^−1^) and B3LYP (− 0.2 kcal mol^−1^) are accurate in calculations following by HF/cc-pVDZ (− 0.48 kcal mol^−1^).

*N*-methylpiperidine and 2-, 3-, 4-methylpiperidine [292] adopt a chair conformation with equatorial substitution. *N*,2- and *N*,3-dimethylpiperidines prefer the diequatorial conformation [292]; the interpretation of the relative stability is similar and has been given previously for methyltetrahydropyrans. The second more stable conformation in dimethylpiperidines is the combination C-Me(ax), N-Me(eq) against C-Me(eq), N-Me(ax). In these molecules C-Me(ax) orientation is preferred over N-Me(ax), because the repulsive 1,3-diaxal interactions are bigger in the last orientation due to the smaller length of C-N bond compared to the C-C bond.

An interesting case arises when the substituent at 2-position of *N*-methylpiperidine is a bulky secondary or tertiary alkyl group, like 2- or 1-adamantyl [293]. In both two molecules the chair conformation N-Me(ax), C-Ad(eq) is by far more stable than the other minima. The interaction between adamantyl and methyl is much more important than the axial preference over equatorial just for the *N*-methyl group that determines the conformational preferences for the 2-alkyl-*N*-methylpiperidines where alkyl is small. Furthermore, in 2-(1-adamantyl)-*N*-methylpiperidine and 2-(2-adamantyl)-*N*-methylpiperidine while the chair conformation N-Me(ax), C-Ad(eq) is the global minimum the second more stable conformation is different between the two molecules. In 2-(1-adamantyl)-*N*-methylpiperidine the N-Me(eq), C-Ad(eq) conformation is the second more stable conformation but in 2-(2-adamantyl)-*N*-methylpiperidine the diaxial conformation is the second more stable! In 2-SnBu_3_-N-Me-piperidine the conformations N-Me(eq), C-SnBu_3_(eq) and N-Me(eq), C-SnBu_3_(ax) are almost isoenergetic. The major reason appears to be a distortion of the conformation in which the C-2-Sn bond is synclinal to the nitrogen lone pair [294].

The largest errors in the conformational energies of the se molecules, in which steric interactions contributed significantly, are observed in the calculations performed using Dreiding, UFF force fields. MM3-96 and MMFF94. These force fields calculate erroneously *eq,eq* instead of the *eq,ax* as the global minimum (with more then 3 kcal mol^−1^ energy difference) in *N*,2-dimethylpiperidine, *N*,3-dimethylpiperidine, *N*,4-dimethylpiperidine. For 2-(2-Ad)-N-Me-piperidine the following methods predict the global minimum with small deviations: MM+ (+ 0.22 kcal mol^−1^), MMX (− 0.11 kcal mol^−1^), MM3-96 (− 0.65 kcal mol^−1^), MMFF94 (+ 0.82 kcal mol^−1^), MM4-08 and HF theories (− 0.43 to − 0.22 kcal mol^−1^), B3LYP (− 0.24 kcal mol^−1^) and MP2 (− 0.10 kcal mol^−1^). In the case of 2-(1-Ad)-N-Me-piperidine all theories, except MM4-08, perdict the global minimum, the biggest errors are made by HF theories (+ 0.85 to + 1.25 kcal mol^−1^), then from MMX (+ 0.51 kcal mol^−1^) while MM+ (− 0.12 kcal mol^−1^), MM3-96 (+ 0.07 kcal mol^−1^), MMFF94 (+ 0.09 kcal mol^−1^), MM3-00 (+ 0.01 kcal mol^−1^), B3LYP (+ 0.10 kcal mol^−1^) and MP2 (+ 0.04 kcal mol^−1^) performed with similar accuracy as regards the reference DLPNO-CCSD(T) value.

**Fig. 17:**
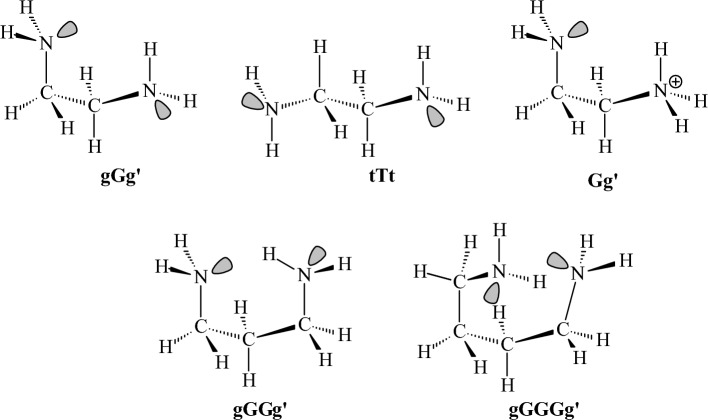
Conformations of diamines H_2_N(CH_2_)_x_NH_2_ (x = 2–4) around N-C-C-N (*G* or *T*) and lp-N-C-C (*g* or *g*+ and *g'* or* g*−) dihedral angles

**Fig. 18:**
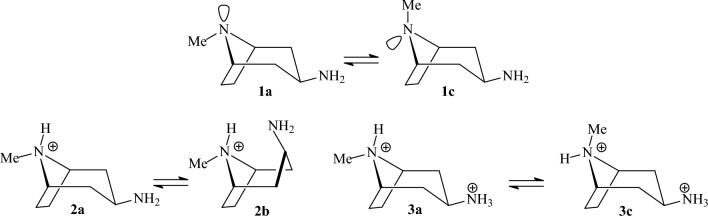
Low energy conformers of β-aminotropane and its cations

Polyamines are interesting molecules because they stimulate ligand binding to the NMDA receptor [295]. Ethanediamine, propanediamine, butanediamine [105] (Fig. 17) and the most stable conformations of β-aminotropane [106] (Fig. 18) are examined. Ιn ethanediamine, propanediamine and butanediamine the conformations *gGg′, gGGg′, gGGGg′* have hydrogen bonding interaction between N-H groups (Fig. 17) [296, 297]. This stabilizing interaction compensates steric repulsion in the case of ethanediamine, propanediamine with *gGg′, gGGg′* more stable over *tTt, tTTTt*, respectively. This is not the case for butanediamine which has *tTTTt* and *gGGGg′* equal in energy. For ethanediamine all the force fields, except UFF and Dreiding, calculate correctly that conformation gGg′, which is stabilized with a hydrogen bonding between N-H groups, is lower in energy than the tTt conformation (Fig. 17); MM+ (0 kcal mol^−1^), MM3-96 (+ 0.04 kcal mol^−1^), MM3-00 (− 0.11 kcal mol^−1^), MM4-08 (− 0.28 kcal mol^−1^) are fairly accurate and performed better that HF theories (− 0.94 to − 0.78 kcal mol^−1^) which also predicted correctly the gGg′ as the global minimum. For propanediamine all the quantum chemistry theories calculate the correct global minimum and from the force fields only MM3-96 predict the global minimum. In the case of butanediamine, only MMFF94 (− 0.11 kcal mol^−1^) showed that *tTTTt* and *gGGGg′* are equal in energy, in agreement with our DLPNO-CCSD(T) calculations. Although MP2 (− 0.02, − 0.23 kcal mol^−1^) and B3LYP (− 0.27, + 0.49 kcal mol^−1^) are accurate as regards ethanediamine and propanediamine, respectively, they performed with a deviation of + 0.24 kcal mol^−1^ and − 0.30 kcal mol^−1^ in the case of propanediamine.

In β-aminotropane there is a six-membered ring and a five-membered ring in the same molecule (Fig. 18). Thus, the conformational preferences [298] of this molecule are determined by the steric interactions between coaxial N-Me group and C-H_ax_ bonds in the six-membered ring (Fig. 18, structure **1c**) or in the five-membered ring (Fig. 18, structure **1a**). Our DLPNO-CCSD(T) calculations show that 1a is the global minimum by 0.39 kcal mol^−1^. UFF shows the highest deviation (+ 7.10 kcal mol^−1^), Dreiding (+ 0.86 kcal mol^−1^), MM3-96 (+ 0.65 kcal mol^−1^) and MM3-00 (+ 0.76 kcal mol^−1^), MMX (+ 0.10 kcal mol^−1^), MM+ (− 0.01 kcal mol^−1^), MMFF94 (− 0.07 kcal mol^−1^), MM4-08 (− 0.01 kcal mol^−1^), HF/cc-pVDZ (+ 0.03 kcal mol^−1^), MP2 (+ 0.14 kcal mol^−1^) calculate accurately this conformational preference while HF/CBS and B3LYP calculate conformations 1a and **1c** having equal energy.

For various ammonium derivatives MP2/6-311G(d,p) and MM3 calculations have been performed [106, 299]. For ethyldimethylamine or [299] *N*-methylpiperidine [292] or the dication of β-aminotropane (relative stability of conformations 3c, 3a) the MMX or MM4-08 or MM+ fail to calculate the correct global minima. MP2 (+ 0.02 kcal mol^−1^, + 0.05 kcal mol^−1^, + 0.07 kcal mol^−1^), B3LYP (+ 0.13 kcal mol^−1^, + 0.36 kcal mol^−1^, − 0.28 kcal mol^−1^), HF/cc-pVDZ (+ 0.15 kcal mol^−1^, + 0.55 kcal mol^−1^, + 0.03 kcal mol^−1^), HF/cc-pVTZ (+ 0.23 kcal mol^−1^, + 0.65 kcal mol^−1^, + 0.03 kcal mol^−1^), HF/CBS (+ 0.33 kcal mol^−1^, + 0.69 kcal mol^−1^, + 0.03 kcal mol^−1^) and MM3-00 (− 0.24 kcal mol^−1^, − 0.50 kcal mol^−1^, + 0.38 kcal mol^−1^) performed with the smaller devations following by MMFF94 (− 0.08 kcal mol^−1^, − 0.15 kcal mol^−1^, − 4.33 kcal mol^−1^) and MM3-00 (− 0.24 kcal mol^−1^, − 0.50 kcal mol^−1^, − 1.23 kcal mol^−1^).

For the monocations of β-aminotropane and ethanediamine, the conformational preferences are determined as a compromise between hydrogen bonding interactions and steric repulsions. The MM+, MMX, UFF, Dreiding, MM4-08 and MM+, UFF, Dreiding fail to calculate the correct global energy minima. MP2 (+ 0.89 kcal mol^−1^, + 0.21 kcal mol^−1^) and B3LYP (+ 0.54 kcal mol^−1^, − 0.27 kcal mol^−1^) theories performed with the smallest devations following by HF/cc-pVDZ with deviation − 2.28 kcal mol^−1^, − 2.97 kcal mol^−1^, respectively, while all other theories deviate more.

Some amides and dipeptides were examined as models to test the accuracy of the tested methods when calculating the relative energies for conformers generated by rotation around the amide CO-N bond [300]. The simpler compounds tested are *N*-methylformamide, *N*-methylacetamide, formamidine [301] and *N*-methylformamidine. There are experimental studies in the gas phase [302–304] and ab initio calculations [305, 285, 288–291] for *N*-methyformamide and experimental studies [285, 286, 290, 292–294, 306] in the gas phase and ab initio calculations [307–309] for *N*-methylacetamide and formamidine [301]. For *N*-methylacetamide other studies show that the enthalpy difference at 298 K is in the 2.1–2.5 kcal mol^−1^ range according to experimental results in the gas phase and in solution [310, 311] or ab initio results in the gas phase [305, 312, 313] or ensemble simulations in solution [314]. The difference diminishes to 1.0–1.3 kcal mol^−1^ for *N-*methylformamide according to DNMR [203] or ab initio calculations [312] due to reduced steric crowding in the *E* form. Our DLPNO-CCSD(T) results calculate these energy differences to be 2.11 kcal mol^−1^ for *N-*methylformamide and 1.08 kcal mol^−1^ for *N-*methylacetamide while for formamidine and *N*-methylformamidine the conformational energies are 1.72 and 1.22 kcal mol^−1^, respectively. All the force fields calculate Z-conformation as the global minimum with Deiding (− 1.37 kcal mol^−1^), UFF (− 1.66 kcal mol^−1^) and MMX (− 1.44 kcal mol^−1^) showing the highest deviation from the DLPNO-CCSD(T) reference value while MP2 (− 0.04 kcal mol^−1^), MM+ (+ 0.07 kcal mol^−1^), MM3-96 (+ 0.07 kcal mol^−1^) and B3LYP (− 0.10 kcal mol^−1^) performed best. For *N*-methyformamide, MMX, UFF and Dreiding inaccurately calculate the *E*-conformation as the global minimum, MM3-00 (+ 0.83 kcal mol^−1^), MM4-08 (+ 0.58 kcal mol^−1^) deviate from the 1.08 kcal mol^−1^ reference value while MP2 (+ 0.04 kcal mol^−1^), HF/CBS (− 0.10 kcal mol^−1^), HF/cc-pVTZ (− 0.11 kcal mol^−1^), HF/cc-pVDZ (− 0.14 kcal mol^−1^), B3LYP (− 0.22 kcal mol^−1^) and MM+ (− 0.25 kcal mol^−1^) performed accurately. For formamidine and *N*-methylformamidine MM+, MMX, MM3-00 and MM4-08 calculate erroneously the *cis* as global minimum and for *N*-methylformamidine the MM3-00 (+ 2.58 kcal mol^−1^) has the largest deviation. For these two molecules MP2 (− 0.01 kcal mol^−1^, + 0.12 kcal mol^−1^) performed the best, while B3LYP performed modestly (+ 0.40 kcal mol^−1^, + 0.49 kcal mol^−1^).

**Fig. 19:**
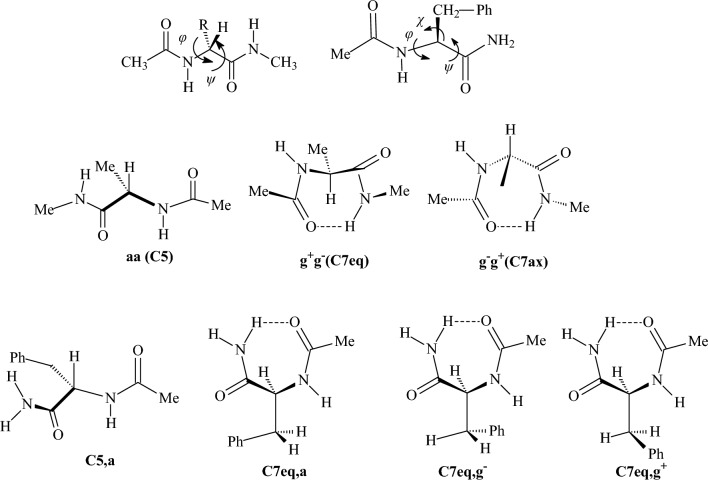
Low energy conformers of *N*-acetylalanine-*N*-methylamide and of *N*-acetylphenylalaninyl-amide (NAPA) by rotation around *φ* (OC-N-C-CO), *ψ* (N-C-CO-N) and *χ* (N-C-C-Cipso) dihedral angles; Ramachandran and IUPAC definitions are used

Double resonance IR/UV and Raman spectroscopy in the gas phase has emerged as a powerful tool for studying conformational preferences of small model peptides containing UV chromophores [315], e.g. measurements of the populations of the three major backbone conformations in 19 amino acid dipeptides using IR and Raman spectroscopy in the gas phase [316, 317]. UV spectroscopy, being sensitive to the chromophore environment, helps identify conformational isomers present in a given sample, while the combination of IR spectroscopy and DFT or ab initio calculations [111, 318–322] allow σ determination of their geometries. The folding processes in peptides are thought to be governed mainly by hydrogen bonding, whose signature in the fingerprint region of the vibrational spectrum enables identification of a specific conformer. The terminal acetyl and amide groups increase the length of the peptide chain by one unit while the series of the aliphatic residues allow one to follow the changes in the conformational preferences [323] of the peptide with increasing size and hydrogen bonding capabilities between N-H and C=O of the residues. Here, we examined the conformations in three model aminoacids with terminal acetyl and amide groups, e.g., *N*-acetylglycine-*N*-methylamide [324, 325], *N*-acetylalanine-*N*-methylamide [320, 326], *N*-acetylphenylalaninylamide (NAPA) [111, 318–321] (Fig. 19). The angles *φ* and *ψ* of the HF/6-31+G* geometry-optimized conformers [111] C7eq, C5 and C7ax are C7eq (− 86, 78), C5 (− 204, 200), C7ax (75, − 54) and for the B3LYP/6-31+G* optimized structure are C7eq (− 83, 73), C5 (− 158, 163), C7ax (73, − 56). These conformations correspond to interactions between the carbonyl and amide groups of the same residue resulting in a formation of a five-membered ring (C5) and leading to the extended β-strand or to interactions between the carbonyl and amide groups of adjacent residues resulting in a seven-membered ring structure C7 or γ-turn [323]. The energies of NAPA conformers have been previously calculated at the CASSCF/MS-CASPT2//B3LYP/6-31+G** level of theory and are also shown in Table 5 [322].

For *N*-acetylglycine-*N*-methylamide, *N*-acetylalanine-*N*-methylamide and NAPA the HF theories inaccurately calculate the relative ranking of the conformations. All force fields calculate correctly the local minima ranking for the two first peptides but MM+, UFF, MM3-96, MM4-08 show the bigger deviations, i.e. > 1.5 kcal mol^−1^ or even 3 kcal mol^−1^. The best performance was observed for MP2 (+ 0.13 kcal mol^−1^, + 0.14 kcal mol^−1^), B3LYP (− 0.66 kcal mol^−1^, − 0.08 kcal mol^−1^) and MMFF94 (− 0.24 kcal mol^−1^, − 0.46 kcal mol^−1^).

For NAPA UFF, Dreiding, MM+, MMX, MM3-00, MM4-08 force fields and HF theories provide an incorrect ranking of the minima with deviation from DLPNO-CCSD(T) reference values > 1.5 kcal mol^−1^ for HF theories and MM+, MMX, Dreiding and > 3 kcal mol^−1^ for UFF force field. MP2 has the best performance following MMFF94 and B3LYP.

### Sulfur- and phosphorus-containing compounds

**Fig. 20:**
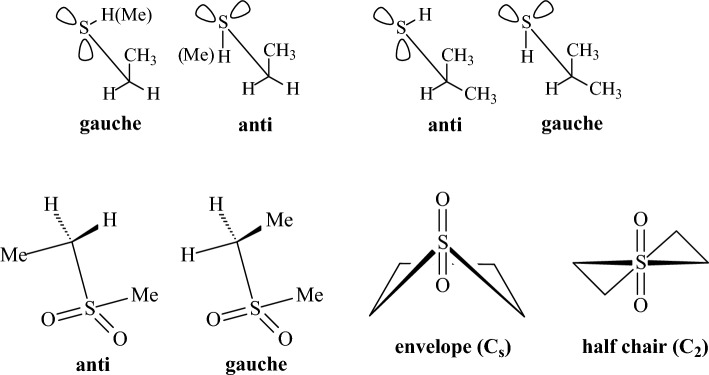
Low energy conformers of EtSH, MeSEt, i-PrSH and of the sulfones MeSO_2_Et and cyclopentanesulfone (sulfolane)

For ethanethiol the *gauche* conformation by rotation around the C-S bond has lower energy than the *anti* conformation, while in 2-propanethiol the *anti* conformation has been suggested that is stabilized slightly over the *gauche* conformation as has been shown by B3LYP 6-311++G(2df, 2pd) calculations for ethanethiol [327] and 2-propanethiol [327] (Fig. 20). These results are confirmed by our DLPNO-CCSD(T) calculations. It was suggested that the Pauli repulsive (steric interactions and hyperconjugative interactions, i.e. *σ*(H-C) → σ*(C-H) or *σ*(H-C) → σ*(C-Me) contribute to the conformational energies. Thus, in the higher in energy *anti* conformation of ethanethiol the destabilization effect of the two *gauche* lone pair-methyl interactions is higher than the stabilization effect of the two *σ*(H-C) → σ*(C-H) hyperconjugative interactions. Also in the *gauche* conformation of ethanethiol there is one destabilizing *gauche* lone pair-methyl interaction and two stabilizing electronic interactions, i.e. the *σ*(H-C) → σ*(C-H) and *σ*(H-C) → σ*(C-Me) hyperconjugative interactions. In methyl ethyl sulfide the presence of one more *gauche* methyl–methyl interaction in *gauche* conformation does not change the conformational energies.

In 2-propanethiol, three lone pair-*gauche* repulsive interactions exist in *gauche* conformation compared to two interactions in the *anti* conformation. For the methyl ethyl sulfone [328, 329], the *anti* conformer with the two methyl groups in *anti* position is more stable than *gauche* conformer with the two methyl groups are crowded in the *gauche* position.

The UFF and Dreiding force fields failed to calculate the *gauche* conformation as the global minimum for ethanethiol. HF/cc-pVDZ (− 0.03 kcal mol^−1^), MP2 (− 0.09 kcal mol^−1^), MM4-08 (− 0.11 kcal mol^−1^) have the smallest deviations following by B3LYP (− 0.19 kcal mol^−1^) while MMX (− 0.19 kcal mol^−1^) shows the biggest deviation. The *gauche* and *anti* conformations are of equal energy in 2-propanethiol according to our reference DLPNO-CCSD(T) calculations and MP2, HF/CBS, HF/cc-pVTZ, MMX agree with this result differing by less than ~ 0.1 kcal mol^−1^ calculations. The MMFF94 (+ 0.64 kcal mol^−1^), MM3-00 (+ 0.49 kcal mol^−1^) show the biggest deviation following by MM3-96 (+ 0.33 kcal mol^−1^), MM4-08 (+ 0.30 kcal mol^−1^) and MM+ (+ 0.32 kcal mol^−1^). For methylethylsulfone MMX, UFF provide erroneously the *gauche* instead of the *anti* conformation as the global minimum. MP2 (− 0.01 kcal mol^−1^), Dreiding (− 0.07 kcal mol^−1^) following by MM4-08 (− 0.11 kcal mol^−1^) and B3LYP (− 0.13 kcal mol^−1^) while the biggest deviations are performed with MMFF94 (+ 1.20 kcal mol^−1^) following by MM+ (+ 0.57 kcal mol^−1^), HF/CBS (+ 0.31 kcal mol^−1^), HF/cc-pVTZ (+ 0.30 kcal mol^−1^), HF/cc-pVDZ (+ 0.26 kcal mol^−1^), MM3-00 (+ 0.21 kcal mol^−1^), MM3-96 (+ 0.21 kcal mol^−1^).

The equatorial position is favoured when thiane ring is substituted with methyl resulting in 2-methyl, 3-methyl or 4-methylthiane [178, 330, 331]. The smaller difference between the axial and equatorial conformers in *S*-methylthiane cation [331–333] and 2-methylthiane [330] compared to that of methylcyclohexane or *N*-methylpiperidine can be attributed to the smaller 1,3-repulsive interactions because of the longer C-S bonds and the opening of S-C-C bond angles. The conformational energies for 4-methylthiane and the twist boat–chair equilibrium for thiacylohexane are similar to that of methylcyclohexane and cyclohexane, respectively. For sulfolane, the five-membered ring prefers the half chair C_2_ conformation compared to the envelope conformation C_s_ [334–337]. MP2 (+ 0.01, − 0.05, + 0.06, + 0.01 kcal mol^−1^, + 0.32, + 0.22 kcal mol^−1^) shows the smallest deviation for all six molecules 2-methyl, 3-methyl or 4-methylthiane, S-methylthianium, thiacyclohexane and sulfolane the tested conformational energies compared to our reference DLPNO-CCSD(T) calculations. The next higher accuracy is achieved by MM+, MMX, MM3-96, MM3-00 force fields for the methylthiane series (+ 0.11 ± 0.28 kcal mol^−1^), by B3LYP for *S*-methylthianium (+ 0.01 kcal mol^−1^), B3LYP (+ 0.32 kcal mol^−1^) and MM3-96, MM3-00 force fields (+ 0.38 kcal mol^−1^) for thiacyclohexane, and for sulfolane by HF/cc-PVTZ (+ 0.23 kcal mol^−1^), HF/CBS (+ 0.17 kcal mol^−1^) and MMX (+ 0.34 kcal mol^−1^).

**Fig. 21:**
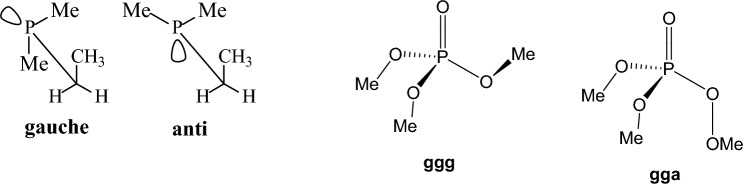
Conformations of ethyldimethylphosphine and trimethoxyphosphate

Ethylphosphine prefers for the C-C-P-lp moiety the *anti* conformation [338] in which the lone pair-methyl steric repulsion is minimized, while the *gauche* conformation is the global minimum in ethyldimethylphosphine [339]. In ethyldimethylphosphine, C-Me prefers the position between the lone pair and P-methyl in *gauche* conformation (Fig. 21) compared to the *anti* conformation having a position between two P-Me groups. Additionally, the hyperconjugative interaction *n*(P) → σ*(C-H) can be important for the stabilization of the *gauche* conformation [339]. Compared with the DLPNO-CCSD(T) calculated conformational energy of 0.49 kcal mol^−1^ for ethylphosphine, the MP2 (+ 0.06 kcal mol^−1^), B3LYP (+ 0.06 kcal mol^−1^), MM3-00 (+ 0.06 kcal mol^−1^), MM3-96 (+ 0.08 kcal mol^−1^), MM4-08 (+ 0.13 kcal mol^−1^), MM+ (+ 0.10 kcal mol^−1^), performed accurately while HF theories deviate from 0.12 to 0.24 kcal mol^−1^ and UFF, Dreiding and MMFF94 calculate *gauche* and *anti* conformations with equal energy. In the case of ethyldimethylphosphine all theories calculate correctly that *gauche* conformation is the global minimum with MP2 (− 0.05 kcal mol^−1^) and MM+ (− 0.06 kcal mol^−1^) showing the smallest deviation following by MMX (− 0.13 kcal mol^−1^), B3LYP (− 0.19 kcal mol^−1^) and MM4-08 (+ 0.13 kcal mol^−1^); more than 1.5 kcal mol^−1^ deviation is shown with MMFF94 and UFF and the largest deviation with Dreiding (− 4.56 kcal mol^−1^).

For the trimethyphosphate the global minimum corresponds to a *ggg* orientation of P=O with the three OMe groups by rotation around the P-O single bond [340–342], as shown by matrix isolation IR and DFT computations, which have been expanded to the higher analogues like tri-*n*-butyl phosphate [343].

Six-membered *N*-alkylphosphiranes prefer the equatorial position from the axial position [344, 345]. However, the conformational energy is much smaller compared to the C-alkyl analogues, i.e. the alkylcyclohexanes or the *N*-alkyl analogues, i.e., the *N*-alkylpiperidines, because the 1,3-diaxal repulsive interactions are smaller due to the longer C-P bonds and the wider P-C-C bond angles, as described also for the thio- or oxa-analogues. MM3-00 [346] and MM4-08 provide incorrect global minimum (i.e., the axial conformation), UFF or Dreiding the largest deviation, greater that 1.5 or 3 kcal mol^−1^, respectively, followed by MM+ (− 0.40 kcal mol^−1^) and HF/cc-pVTZ (+ 0.36 kcal mol^−1^), HF/CBS (+ 0.45 kcal mol^−1^). The most accurate results are observed with MP2 (+ 0.06 kcal mol^−1^), following by HF/cc-pVDZ (+ 0.11 kcal mol^−1^) and B3LYP or MMX (+ 0.21 kcal mol^−1^).

### Conjugated compounds

For 1,3-butadiene the energy difference between the *gauche* (*cis*) form and ground state *trans* form was determined in the gas phase using Raman [347] or microwave [348] or UV [349] spectroscopy to be 2.94 kcal mol^−1^ which agrees well with the ~ 3.01 kcal mol^−1^ from MP2/aug-cc-pVTZ [350] or very accurate CCSD(T)(FC)/CBS + CCSD(T)(CV)/cc-pwCVQZ + scalar relativistic effects correction + CCSDT(Q)(FC)/cc-pVDZ correction [351] (Table 7). The first microwave spectrum of “*cis*” butadiene unambiguously shows that it possesses a nonplanar *gauche* structure [348]. Acrolein has been studied experimentally in the gas phase [352] and with ab initio calculations at the CCSD(T)/CBS level of theory [353] providing energies 2.20 kcal mol^−1^ and 2.06 kcal mol^−1^, respectively. Methacrolein has been studied experimentally in the gas phase [354] and with ab initio calculations at the CCSD(T)/CBS [355] providing energies 3.02 kcal mol^−1^ and 3.47 kcal mol^−1^, respectively. Methyl vinyl ketone has been studied experimentally in the gas phase [356] and with ab initio calculations at the CCSD(T)/CBS [355] providing energies 0.80 kcal mol^−1^ and 0.61 kcal mol^−1^, respectively. Our results are in good agreement with values provided by CCSD(T)/CBS.

In the case of methyl vinyl ketone, UFF, Dreiding and HF/cc-pVDZ failed to calculate the *trans* conformation as the ground state. In all other cases the theories tested calculated the correct global minimum. MP2 is the most accurate following B3LYP for 1,3-butadiene, B3LYP and MMFF94 for acrolein, MMFF94 for methyl vinyl ketone. Among force fields, MMFF94 is the best performer while from Allinger’s force fields it is MM4-08, which is better parameterized for conjugated compounds [357].

### Energy barriers

**Fig. 22:**
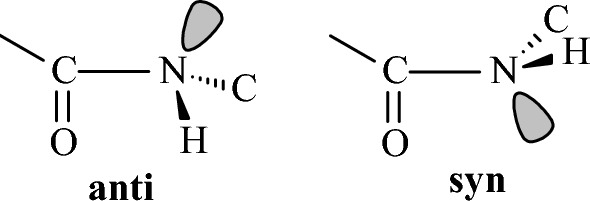
Possible structures for the transition state by rotation around CO-N bond

The results of the calculations and the experimental data for some conformational energy barriers including C-C, C-O, C-N, C-S, C-P and CO-N are shown in Table 8. The transition state for the rotation around a C-C bond corresponds to the conformer (a) with eclipsed C-H bonds in ethane [358], (b) with eclipsed C-C and C-H bonds in propane [359], and (c) with eclipsed the two C-C bonds in butane [360]. The energy demanded C-C bond rotation was also calculated for (1-adamantyl)-1-methyl-ethylchloride, 1-(tert-butyl)-1-methyl-ethylchloride and in 1-(bicyclooctyl)-1-methyl-ethylchloride [361]. Similarly, for the C-C bond rotation in ethanol [362], ethylamine [363], and ethanethiol [364], the transition state involves eclipsing of the C-H and C-X(sp^3^) bonds (X = OH, NH_2_ or SH respectively). Among the molecules of Table 8, rotation around C(sp^3^)-C(sp^2^) bonds was examined for propene [365] and acetone [366]. In the transition state, C-H and C = X (X = O or CH_2_) bonds have a gauche orientation. The C-X bond rotation was calculated for some model molecules including methylamine, methanol [367], dimethylether [368], methyl formate [109], methanethiol [369], dimethylsulfide [370–372], dimethylsulfone [373], dimethylphosphine [110], and trimethylphosphate [346]. For the CO-X bond rotation (X = O, N) we examined methyl formate and methylacetamide. In the transition state of methyl formate [81] the O=C-O-C dihedral angle is 90° while in methylacetamide between the two likely transition states shown in Fig. 22 the anti conformer is preferred [374, 375].

**Fig. 23:**
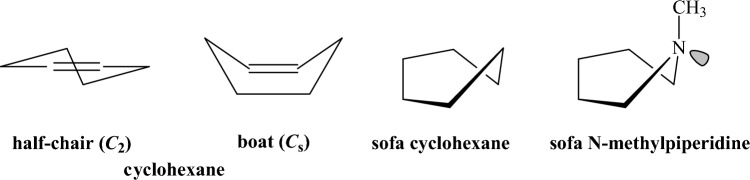
Ring inversion transition states for cyclohexane, cyclohexene and *N*-methylpiperidine

The transition states for ring and nitrogen inversion were investigated for some systems. The structure of the transition state for the ring inversion in cyclohexane [77, 376], cyclohexene [141], and *N*-methylpiperidine [377] are shown in Fig. 23. For *N*-methylpiperidine, *N*-methylpyrrolidine and 3,3-dimethyl-*N*-pyrrolidine [378] nitrogen inversion transition has a planar nitrogen configuration.

MP2 best performed with calculated values close to the reference with exception of C-P bond rotation in dimethyl phosphine. It has been reported that semilocal DFT potentials including the DFT-HF hydrid methods such as B3LYP can perform well for rotational of conformational barriers [379]. B3LYP performed fairly but in many cases other theories performed more accurately, e.g., MMX, MM+, MM3-96 [79–81], MM3-00 or HF/cc-pVDZ; even Dreiding and UFF performed well in a few cases. Among the force fields, the MM3-00 and MM4-08 deviate the least from the reference values.

## Conclusions

In the present work we revisited previous works assessing the accuracy of force fields as regards the conformational preferences and energies of reference organic molecules. We calculated 158 conformational energies and barriers from 145 organic molecules, including hydrocarbons, haloalkanes, conjugated compounds, and oxygen-, nitrogen-, phosphorus- and sulphur-containing compounds. We reviewed in detail the conformational aspects of these model organic molecules providing the current understanding of the steric and electronic factors that determine the stability of low energy conformers and the literature including previous experimental observations and calculated findings. The suitable energies for comparison with CC-calculated conformational energies are energies measured in the gas phase with spestroscopic methods. Compared to previous work [48, 50], we increased the number of tested molecules and the number of methods applied. We used the UFF and DREIDING force fields, the Allinger’s force fields MM3-96, MM3-00, MM4-80, the MM2-91 clones MMX and MM+, the MMFF94 force field, ab initio theories, e.g. HF, the low-order post-HF MP2 method and the standard DFT model B3LYP. As reference conformational energy values to test the accuracy of these theories we performed basis-set extrapolated DLPNO-CCSD(T) calculations. This enabled us to have a common high-level reference for all compounds and all energetic quantities used in this work, compared to previous studies which often used inconsistent experimental values or low theory levels as reference values.

**Fig. 24:**
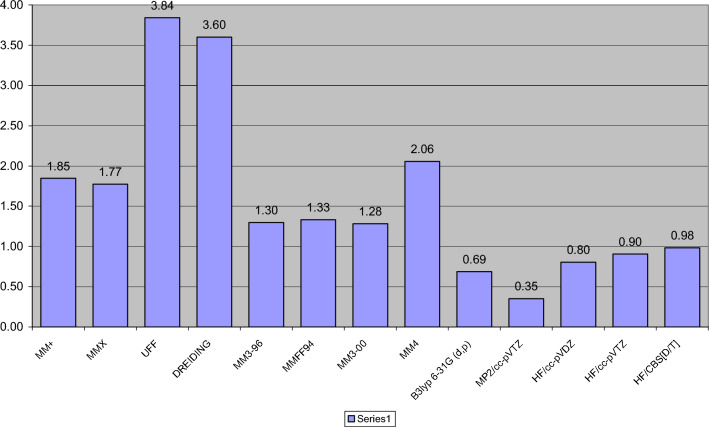
Comparative performance (mean error in kcal mol^−1^) of different theories using 158 conformational energies and barriers from 145 standard organic molecules compared to the DLPNO-CCSD(T)/cc-pVTZ calculated values

As shown in Fig. 24, the lowest mean error value was calculated for MP2 (0.35 kcal mol^−1^), followed by B3LYP (0.69 kcal mol^−1^) and the HF theories (0.81–1.0 kcal mol^−1^). As regards the force fields the lowest errors were observed for the Allinger's force fields MM3-00 (1.28 kcal mol^−1^), ΜΜ3-96 (1.40 kcal mol^−1^) and the Halgren’s MMFF94 force field (1.30 kcal mol^−1^) and then for the MM2-91 clones MMX (1.77 kcal mol^−1^) and MM + (2.01 kcal mol^−1^) and MM4-08 (2.05 kcal mol^−1^). The MM4-08 force field’s lower performance is of some interest but is likely consistent with the effort of Allinger and colleagues to build a set of parameters that might be more useful for vibrational data. The DREIDING (3.63 kcal mol^−1^) and UFF (3.77 kcal mol^−1^) force fields have the lowest performance.

At this point, it is necessary to point out the dramatically different computational cost of the methods compared in this study. The present work considers three distinct categories of computational methods: force-field based molecular mechanics approaches, self-consistent-field QM methods (HF and DFT), and correlated wave-function methods (MP2 and DLPNO-CCSD(T)). Although the results and the errors are presented and discussed in common, it is important to keep in mind that the computational cost of each class of method differs by approximately an order of magnitude or more. Thus, molecular mechanics calculations for even the largest molecules in the present work are completed in time scale of seconds, HF and DFT calculations within several minutes, while the most expensive DLPNO-CCSD(T) calculations may require tens of minutes to a few hours to complete for the largest compounds. The heightened sensitivity of the ab initio quantum chemical methods and their non-linear scaling with respect to the basis set size is an additional consideration that does not apply to molecular mechanics methods. Moreover, in addition to increased time requirements, the correlated wave function methods have much steeper scaling memory/storage requirements with increasing size of the molecule or basis set. These considerations make it impossible to establish cost/error relationships for the whole variety of methods examined herein. Although the abovementioned order-of-magnitude cost comparison should always be considered, each class of method has its own scope, and often a combination of methods with different accuracy/cost profile can be beneficial in practice. Therefore, the choice of method in actual applications should consider not simply the average error and expected accuracy of any given method, but also the substantially different time and memory requirements, the total number of calculations required (e.g., a small set of compounds or a library of thousands of compounds), and the purpose of the study (e.g., rapid screening or benchmark-quality results).

Overall, the current study reviewed and commented on the current state of the art as regards the conformational energies of model organic molecules often present in drug-like molecules and provides a new data set with DLPNO-CCSD(T) calculated values that can be used in future evaluation of approximate computationally efficient methodologies, or even in the training and parameterization of refined force fields.

### Supplementary Information

Below is the link to the electronic supplementary material.

Supplementary file1 (RAR 194 KB)

## Data Availability

Files with structures and data produced with the different calculations methods can be found in following the link: https://github.com/ankolocouris/dlpno_extrapolated_dt. And there is also available the sdf file with all tested organic molecules structure outputs.”
