# Correction to: Conformational energies of reference organic molecules: benchmarking of common efficient computational methods against coupled cluster theory

**DOI:** 10.1007/s10822-023-00531-3

**Published:** 2023-09-29

**Authors:** Ioannis Stylianakis, Nikolaos Zervos, Jenn-Huei Lii, Dimitrios A. Pantazis, Antonios Kolocouris

**Affiliations:** 1 Department of Medicinal Chemistry, Faculty of Pharmacy, National and Kapodistrian University of Athens, Panepistimioupolis Zografou, Athens, 15771 Greece; 2 Department of Chemistry, National Changhua University of Education, Changhua City, Taiwan; 3 Max-Planck-Institut für Kohlenforschung, Kaiser-Wilhelm-Platz 1, 45470 Mülheim an der Ruhr, Germany; 4 Laboratory of Medicinal Chemistry, Section of Pharmaceutical Chemistry, Department of Pharmacy, National and Kapodistrian University of Athens, Panepistimioupolis Zografou, Athens, 15771 Greece

**Correction to: Journal of Computer-Aided Molecular Design**

10.1007/s10822-023-00513-5

In the original article, the Data availability statement was missing and should have read as below.

‘Data availability

Files with structures and data produced with the different calculations methods can be found in following the link:

https://github.com/ankolocouris/dlpno_extrapolated_dt. And there is also available the sdf file with all tested organic molecules structure outputs.’

The original article has been corrected.

